# Diagnostic Approaches and Surgical Outcomes in Nasal Valve Dysfunction: A Systematic Review

**DOI:** 10.3390/diagnostics16091324

**Published:** 2026-04-28

**Authors:** Mahmoud Daoud, Luana-Maria Gherasie, Maria Louise Fufezan, Răzvan Hainăroșie, Cătălina Voiosu, Andreea Rusescu, Irina-Gabriela Ioniță, Oana-Ruxandra Aliuș, Viorel Zainea

**Affiliations:** 1 Faculty of Medicine, “Carol Davila” University of Medicine and Pharmacy, 050474 Bucharest, Romania; mahmoud.daoud@drd.umfcd.ro (M.D.);; 2 Department of Otolaryngology and Head and Neck Surgery, Cronos Med Medical Center, 014131 Bucharest, Romania; 3 Department of Functional ENT Surgery, “Prof. Dr. D. Hociotă” Institute of Phonoaudiology and Functional ENT Surgery, 050751 Bucharest, Romania; 4 Department of Plastic and Reconstructive Surgery, Bucharest Emergency Clinical Hospital, 014461 Bucharest, Romania

**Keywords:** nasal valve dysfunction, nasal airway obstruction, nasal valve collapse, rhinomanometry, acoustic rhinometry, NOSE score, functional rhinoplasty, nasal valve surgery

## Abstract

**Background:** Nasal valve dysfunction (NVD) is a common yet underrecognized cause of nasal airway obstruction, with a significant impact on quality of life. Despite its clinical relevance, no universally accepted diagnostic standard exists, and optimal management remains debated. Multiple diagnostic tools and surgical or minimally invasive treatments have been proposed. This systematic review and meta-analysis aimed to evaluate current evidence regarding diagnostic approaches and treatment outcomes in NVD. **Methods:** A systematic search of PubMed/MEDLINE, Embase, and Cochrane Library was performed for studies published between January 1990 and January 2026, in accordance with PRISMA 2020 guidelines. Randomized controlled trials, non-randomized comparative studies, cohort studies, and case series (≥10 patients) assessing diagnostic methods or therapeutic interventions for NVD were included. Diagnostic data were synthesized narratively. The primary surgical outcome was change in the Nasal Obstruction Symptom Evaluation (NOSE) score. Risk of bias was assessed using RoB 2, ROBINS-I, and QUADAS-2 tools. **Results:** Seventy-two primary clinical studies were included (15 diagnostic, 57 treatment-focused). Objective airflow measurements, particularly rhinomanometry and peak nasal inspiratory flow, showed greater reliability than isolated clinical maneuvers. Imaging modalities provided anatomical detail but correlated inconsistently with symptoms. Meta-analysis of 12 studies (*n* = 1210 patients) suggests that both traditional surgical and minimally invasive interventions can substantially improve nasal breathing, with mean NOSE score reductions of 40–55 points, though heterogeneity precludes direct comparison of their relative effectiveness. **Conclusions:** Diagnosis of NVD requires a multimodal approach combining clinical assessment, validated symptom scores, and selective objective testing. Surgical and minimally invasive treatments provide substantial symptom improvement when appropriately indicated. Evidence is constrained by the predominance of observational data, emphasizing the need for standardized diagnostics and robust comparative trials.

## 1. Introduction

NVD is defined as a reduction in nasal airflow due to compromise of the nasal valve region, which is the narrowest segment of the nasal airway. Even minor anatomic variations or weaknesses in this area can cause significant increases in airway resistance and symptomatic nasal obstruction, clinically observed as lateral wall collapse on inspiration. NVD may involve the internal nasal valve (the angle between the upper lateral cartilage and septum, normally ~10–15°) and/or the external nasal valve (the lateral ala and nostril margin). Common etiologies include prior rhinoplasty or trauma leading to structural weakness, scarring, septal deviation, congenital cartilage absence, and inflammatory causes like turbinate hypertrophy.

### 1.1. Diagnostic Methods for Nasal Valve Dysfunction

There is currently no single “gold standard” test to confirm valve dysfunction. During physical examination, the structural narrowing of the nasal valve area and a positive Cottle sign (subjective improvement when lateral wall is manually widened) may suggest valve compromise. A modified Cottle maneuver (supporting different areas of the nasal sidewall or lateral crus) has been proposed as a better alternative. Nasal endoscopy allows direct visualization of the nasal valve region and can capture dynamic collapse during inspiration. In selected cases, drug-induced sleep endoscopy (DISE) has been proposed for the evaluation of dynamic collapse that may not be evident during awake examination.

Objective measurements of nasal airflow and resistance support the diagnosis of NVD. Rhinomanometry is considered a standard for measuring nasal airway resistance by recording pressure and flow during respiration. Four-phase rhinomanometry provides accurate analysis of inspiratory vs. expiratory cycles in real time [1]. However, rhinomanometry requires specialized equipment, a controlled environment (to account for temperature and humidity), and patient cooperation. Despite these limitations, it remains a valuable research tool. Acoustic rhinometry, using sound wave reflections to map the cross-sectional area of the nasal cavity as a function of distance from the nostrils, provides measures of minimal cross-sectional area (MCA) and volume within the nasal passage. Quick and non-invasive, in the context of NVD, acoustic rhinometry can confirm an MCA < 0.5–0.6 cm^2^ at the valve area, indicating significant internal valve compromise. However, like rhinomanometry, acoustic rhinometry does not directly assess how the nasal wall behaves dynamically during inspiration, when collapse may occur. Moreover, its readings can be influenced by technique and anatomy, being less accurate in posterior regions of the nasal cavity. A 2019 European position paper on rhinology diagnostic tools concluded that acoustic rhinometry and rhinomanometry are the most reliable objective tests for nasal obstruction, whereas a simple Cottle test is least specific [2].

Computed tomography (CT) is used to accurately delineate the bone and cartilage anatomy of the nasal valve. CT measurements of valve angles or cross-sectional areas can be made; for instance, a narrow internal valve angle (<10°) on CT might indicate structural NVD. The 2010 AAO-HNS panel clinical consensus states that radiographic studies are rarely helpful for NVD and that, although objective measures exist, they were not widely accepted or routinely used at that time [3]. Since 2010, advances such as four-phase rhinomanometry and Computational Fluid Dynamics (CFD) have enhanced diagnostic precision. CFD uses patient-specific nasal anatomy data from CT/MRI to simulate airflow, air pressure, and heat exchange in the nasal cavity. CFD studies in NVD have revealed that a reduced airflow in the middle meatus region, as occurs with a deviated septum or valve collapse, can lessen mucosal cooling and thereby subjectively feel obstructive [4]. CFD-derived metrics, such as regional resistance and wall shear stress, have shown a moderate correlation with symptom scores.

The Visual Analogue Scale (VAS) and the Nasal Obstruction Symptom Evaluation (NOSE) scale are subjective assessments of nasal airway function. The latter has become the standard outcome measure in functional nasal surgery studies due to its ease of use and validity. The NOSE scale has an established minimal clinically important difference of 30 points, and scores ≥ 55 typically indicate severe/extreme obstruction. Therefore, in a patient with a high NOSE score presenting minimal septal deviation or turbinate hypertrophy, valve dysfunction may be suspected.

### 1.2. Surgical Treatment Methods for Nasal Valve Dysfunction

Traditional functional rhinoplasty techniques targeting the nasal valve have been widely reported to improve nasal obstruction symptoms. Common approaches include spreader grafts for internal nasal valve dysfunction and alar batten, alar rim, or lateral crural strut grafts for external valve insufficiency. Interventions addressing both internal and external components may offer greater functional benefit than isolated techniques. Adjunctive procedures, such as septoplasty and inferior turbinate reduction, are frequently performed when contributing anatomical factors, such as septal deviation or turbinate hypertrophy, are present and may enhance overall outcomes.

More recently, minimally invasive approaches—including bioabsorbable lateral wall implants and temperature-controlled radiofrequency remodeling—have emerged as alternative treatment options. These techniques aim to improve nasal airflow with lower procedural morbidity and may be particularly relevant for selected patients who are not candidates for, or prefer to avoid, traditional surgical intervention.

### 1.3. Study Objective

This systematic review aims to describe and synthesize current diagnostic methods for NVD based on the available evidence, while reflecting contemporary clinical practice to inform therapeutic decision-making. The primary research question is “In patients with suspected nasal valve dysfunction, what diagnostic methods are used, and what evidence supports their diagnostic performance?”. Secondly, we ask “Which surgical interventions are indicated for nasal valve dysfunction, and what outcomes reported in the literature support their use?”.

## 2. Methods

### 2.1. Study Design and Reporting Standards

This is a systematic review and meta-analysis, adhering to the Preferred Reporting Items for Systematic Reviews and Meta-Analyses (PRISMA) 2020 statement (Supplementary Tables S1 and S2).

### 2.2. Protocol and Registration

A predefined review protocol was developed prior to study selection and was registered in PROSPERO-registration number CRD420261351284. No amendments were made to the registered protocol subsequent to study commencement.

### 2.3. Eligibility Criteria

Table 1 summarizes the predefined inclusion and exclusion criteria applied during the PRISMA-compliant screening process. Criteria were established to ensure the selection of clinically relevant studies evaluating diagnostic approaches and surgical or minimally invasive treatments for nasal valve dysfunction, with emphasis on appropriate study design, patient population, intervention type, and outcome reporting.

### 2.4. Information Sources and Search Strategy

A systematic literature search was performed in PubMed/MEDLINE, Embase and Cochrane Library by two independent reviewers. The search was last updated in January 2026. The search strategy combined controlled vocabulary and free-text terms related to nasal valve pathology, diagnostic assessment, and treatment modalities. Two separate search strategies were employed: one for diagnostic approaches and another for surgical outcomes of nasal valve dysfunction. The following search strings were employed in the advanced search engines of the above-mentioned databases:

Diagnostic approaches:
(“nasal valve dysfunction” OR “nasal valve collapse” OR “lateral wall insufficiency”) AND (“nasal valve” OR “internal nasal valve” OR “external nasal valve”) AND (rhinomanometry OR “acoustic rhinometry” OR “peak nasal inspiratory flow” OR PNIF OR “computational fluid dynamics” OR “NOSE score” OR “nasal obstruction symptom evaluation” OR Cottle OR “nasal endoscopy” OR “computed tomography” OR CT OR VAS OR “Visual Analogue Scale”) AND (specificity OR sensitivity).
Surgical outcomes:
(“nasal valve dysfunction” OR “nasal valve collapse” OR “lateral wall insufficiency”) AND (“nasal valve” OR “internal nasal valve” OR “external nasal valve”) AND (“spreader graft” OR “alar batten graft” OR graft OR rhinoplasty OR septorhinoplasty OR “nasal valve repair” OR radiofrequency OR “bioabsorbable implant”).

Reference lists of included studies and relevant reviews were manually screened to identify additional eligible publications.

### 2.5. Study Selection

All retrieved records were imported into a reference management system, and duplicate entries were removed. Two reviewers independently screened titles and abstracts for relevance. Full-text articles were subsequently assessed for eligibility based on the predefined inclusion and exclusion criteria. At full-text stage, studies were excluded for reasons including wrong population, non-clinical design, insufficient sample size, lack of relevant outcomes, and inability to isolate nasal valve dysfunction from concurrent sinonasal procedures. A list of excluded studies with reasons is provided in Supplementary Table S3.

**Figure 1:**
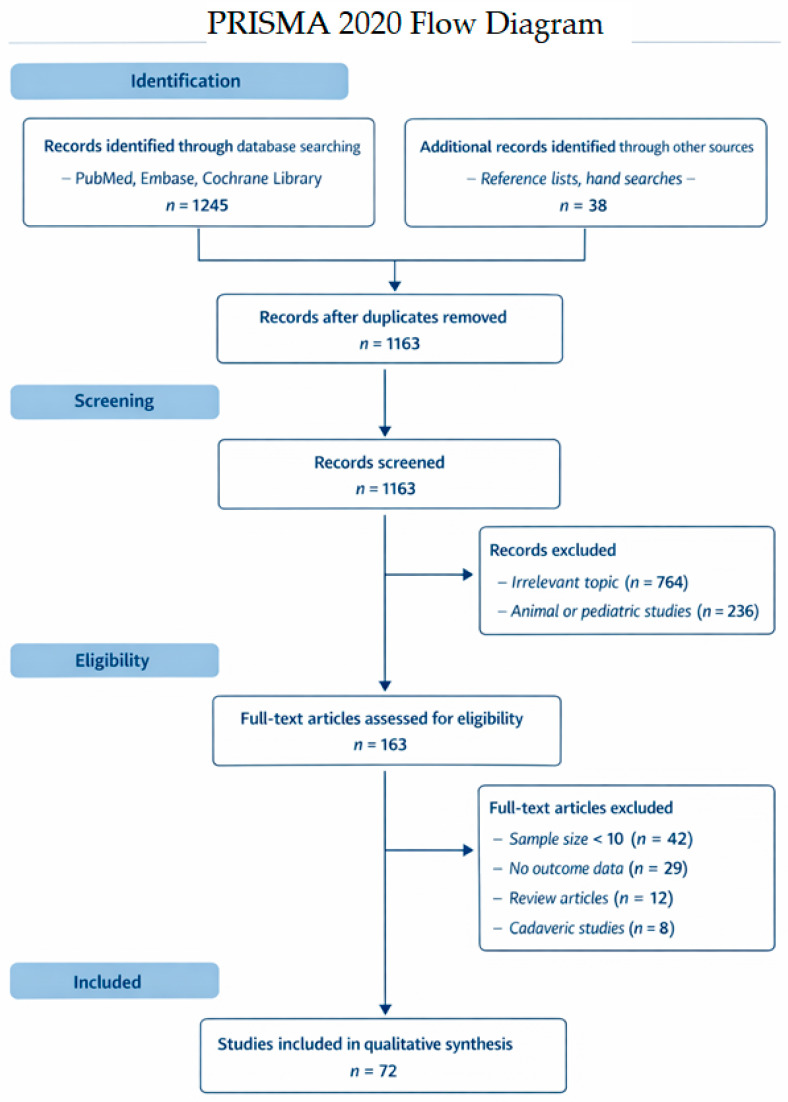
PRISMA 2020 flow diagram of study selection. The diagram illustrates the systematic identification, screening, eligibility assessment, and inclusion of studies evaluating diagnostic approaches and surgical outcomes in nasal valve dysfunction.

Disagreements were resolved by consensus. The study selection process was documented using a PRISMA 2020 flow diagram (Figure 1).

### 2.6. Data Extraction

Data extraction was performed independently by two reviewers using a standardized data collection form. Extracted data included study characteristics (author, year, country, design), sample size and patient demographics, type of nasal valve dysfunction (internal, external, or combined; dynamic or fixed), diagnostic methods or surgical interventions, concomitant procedures, duration of follow-up, and reported outcomes. Outcomes comprised subjective measures (NOSE score, VAS), objective airflow assessments (PNIF/NPIF, rhinomanometry, acoustic rhinometry), and complication and revision rates. For studies included in the quantitative synthesis, baseline and follow-up mean NOSE scores and standard deviations were extracted; when change scores were not reported, they were calculated from available data.

### 2.7. Outcomes

The primary outcome for quantitative synthesis was the change in Nasal Obstruction Symptom Evaluation score (ΔNOSE) from baseline to postoperative follow-up. A clinically meaningful improvement was defined as a ≥30-point reduction in NOSE score, consistent with established minimal clinically important difference thresholds.

Secondary outcomes included change in VAS for nasal obstruction, objective airflow changes (PNIF, rhinomanometry), acoustic rhinometry parameters, postoperative complications and need for revision surgery.

### 2.8. Risk of Bias Assessment

Methodological quality was assessed according to study design using validated tools: the Cochrane Risk of Bias 2 (RoB 2) tool for randomized controlled trials, the ROBINS-I tool for observational and non-randomized studies, and the QUADAS-2 tool for diagnostic accuracy studies. Risk of bias assessments were performed independently by two reviewers, with discrepancies resolved through discussion.

### 2.9. Data Synthesis and Statistical Analysis

A narrative synthesis was performed for diagnostic modalities due to heterogeneity in reference standards and outcome reporting. Quantitative synthesis was undertaken for surgical outcomes when at least three studies reported comparable NOSE score data. Meta-analysis of NOSE change scores (ΔNOSE) was performed using the random-effects DerSimonian–Laird method, accounting for between-study heterogeneity. When ΔNOSE were not directly reported, they were calculated as the difference between postoperative and baseline means. When required, standard deviations of change were estimated using standard statistical methods based on available data, assuming a conservative correlation coefficient (r = 0.5). When variance data were missing, they were derived from available statistics (confidence intervals, *p*-values, or standard errors) when possible. If these were not available, the studies were excluded from quantitative synthesis. Study weighting was performed using the inverse variance method. Heterogeneity was assessed using the I^2^ statistic. Sensitivity analyses were conducted by excluding studies at high risk of bias. Given the limited number of randomized studies, publication bias was assessed qualitatively rather than by funnel plot analysis. Statistical analyses were performed using Review Manager (RevMan, version 7) and Stata (Release 18).

**Figure 2:**
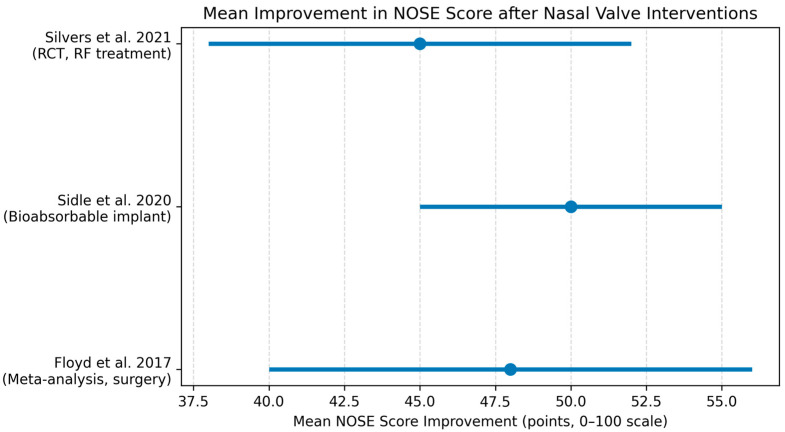
Forest plot of mean improvement in NOSE score (0–100 scale) after nasal valve interventions from selected studies. Patients undergoing traditional functional rhinoplasty [17], and those treated with bioabsorbable lateral wall implants [18] or radiofrequency ablation [19,20] each separately show substantial ability for symptom improvement (~45–50-point NOSE reduction on average). Owing to clinical and methodological heterogeneity, findings represent overall treatment-associated improvement rather than comparative effectiveness. Error bars indicate 95% confidence intervals for the mean change in each study. Individual study-level risk of bias assessments are presented in Supplementary Figure S1.

Results of individual diagnostic studies are summarized in a structured table (Table 2). Results of individual surgical studies are visually represented in a forest plot (Figure 2). Narrative descriptions of individual study findings are provided in Section 3.5.

## 3. Results

### 3.1. Study Selection and Characteristics

The systematic literature search yielded 1245 records across PubMed/MEDLINE, Embase, and the Cochrane Library. After removal of duplicate records and title/abstract screening, 163 full-text articles were assessed for eligibility. Following application of predefined inclusion and exclusion criteria, 72 primary clinical studies were included in the qualitative synthesis (Table 3).

The included studies were published between 1994 and 2025 and originated from 15 countries, reflecting broad international experience in the evaluation and management of NVD. Of these, 15 studies focused on diagnostic evaluation, 57 studies investigated surgical or minimally invasive treatment, and a subset of studies addressed both diagnostic assessment and treatment outcomes.

#### 3.1.1. Diagnostic Studies

The diagnostic studies (*n* = 15) consisted of 3 prospective diagnostic accuracy studies, 2 retrospective observational studies, and 10 cross-sectional or correlation studies examining the relationship between diagnostic modalities and clinical or functional outcomes. Diagnostic approaches included rhinomanometry (6 studies), acoustic rhinometry (4 studies), peak nasal inspiratory flow (3 studies), computed tomography or magnetic resonance imaging (2 studies), computational fluid dynamics (2 studies), and clinical maneuvers such as the Cottle or modified Cottle test (3 studies).

Reference standards varied substantially and included expert clinical assessment, nasal endoscopy, imaging findings, or postoperative response to nasal valve intervention.

#### 3.1.2. Treatment Studies

The treatment studies (*n* = 57) included 1 multicenter randomized controlled trial, 5 non-randomized comparative or cohort studies, and 51 observational case series (Level IV evidence). Sample sizes ranged from small cohorts of 10–20 patients to larger observational datasets. Surgical and minimally invasive techniques were heterogeneous and included spreader grafts (30 studies), alar batten grafts (20 studies), alar rim or lateral crural strut grafts (10 studies), suspension or flaring sutures (5 studies), butterfly grafts (3 studies), and tip rotation or lateral crural repositioning techniques (4 studies). Most studies reported combined approaches, frequently performed in conjunction with septoplasty and/or inferior turbinate reduction.

Follow-up duration ranged from 3 months to 5 years, with the majority of studies reporting outcomes at approximately 12 months. The most frequently reported outcome measures were the Nasal Obstruction Symptom Evaluation (NOSE) score (25 studies), visual analogue scale (VAS) scores (15 studies), clinician-based obstruction grading (10 studies), and objective airflow measurements such as rhinomanometry or PNIF (12 studies). Seven studies included a comparator or control group (e.g., septoplasty alone or sham intervention).

### 3.2. Diagnostic Modalities: Performance and Clinical Utility

Table 3 summarizes the key diagnostic modalities for NVD and the available quantitative performance data.

#### 3.2.1. Clinical Examination and Functional Maneuvers

Clinical examination remained the primary diagnostic approach across studies. The traditional Cottle maneuver demonstrated high sensitivity but very low specificity, limiting its value as a standalone diagnostic test. Several studies reported a high false-positive rate in asymptomatic individuals. In contrast, the modified Cottle maneuver, when applied to specific anatomical regions of the nasal valve, showed improved clinical utility by aiding localization of internal versus external valve dysfunction and informing surgical planning. Das and Spiegel showed that 98% of healthy individuals reported improved airflow with this maneuver, despite having no true obstruction, indicating an extremely high false-positive rate [5]. Fung et al. reports that the modified Cottle exam could correctly identify whether the internal or external valve was the major contributor, thereby guiding the choice of surgical technique [6]. In their cohort, patients selected in this manner showed significant postoperative improvement in breathing, and preoperative modified Cottle results correlated strongly with surgical outcomes. A 2019 survey of Canadian otolaryngologists revealed that the most commonly used diagnostic maneuver was the Cottle test (used by ~63% for external valve assessment) despite its known limitations [21]. Visual inspection of the internal valve area (with or without endoscope) was also frequently used (~40%), whereas objective tests were used far less often [21]. This heterogeneity reflects the lack of a standardized diagnostic protocol for NVD, as also noted by a recent comprehensive review [6,21].

#### 3.2.2. Objective Airflow Measurements

Peak nasal inspiratory flow (PNIF/NPIF) was frequently used as a simple and inexpensive screening tool. Studies consistently reported that lack of PNIF improvement following topical decongestion suggested fixed structural obstruction, including nasal valve dysfunction, whereas significant improvement favored mucosal causes of obstruction. Moreover, a very low PNIF at baseline (e.g., <60 L/min) that normalizes to >120 L/min with a nasal valve dilator in place strongly suggests true valve obstruction that is surgically correctable [2]. PNIF values, on the other hand, were influenced by patient effort and pulmonary function, limiting their diagnostic specificity. Four-phase rhinomanometry demonstrated the highest diagnostic performance among objective tests, with reported sensitivity and specificity approaching 90% for detection of internal nasal valve collapse in selected studies. Nevertheless, limited availability, technical complexity, and variable correlation with patient-reported symptoms restricted its routine clinical use. Acoustic rhinometry reliably identified anatomical narrowing at the nasal valve but provided static measurements that did not consistently correlate with symptom severity or dynamic collapse, serving primarily as an adjunct to anatomical assessment rather than functional diagnosis. A long-term study by Toyserkani et al. found no significant correlation with NOSE scores preoperatively, at 3 months, or at 11-year follow-up [8].

#### 3.2.3. Endoscopy, Imaging, and Advanced Techniques

Nasal endoscopy was considered an essential adjunct to clinical examination. Endoscopic grading systems demonstrated moderate interobserver reliability and correlated with postoperative improvement in selected studies. Erickson et al. used a validated endoscopic grading of the internal nasal valve area before and after surgery, finding that the collapse grade significantly improved after functional rhinoplasty with spreader grafts [10]. There is no standardized endoscopic scoring for NVD akin to NOSE for symptoms. CT or MRI volumetric analysis can estimate total nasal cavity volume, but studies have shown inconsistent correlations between static volumetric measures and patient symptoms. One study found that an automated CT-derived MCA correlated better with NOSE scores than acoustic rhinometry-derived area, suggesting imaging can detect anatomical causes of obstruction [23]. Computational fluid dynamics studies suggested improved correlation between airflow distribution and patient perception of nasal patency; however it remains primarily a research tool due to the computational demands and expertise required. In the future it may help identify the functional impact of subtle anatomic irregularities and guide personalized surgical planning [14].

### 3.3. Surgical Outcomes: Quantitative Synthesis

A subset of 12 studies reporting extractable pre- and postoperative NOSE score data (total *n* = 1210 patients) was included in the quantitative synthesis. Across studies, the mean improvement in NOSE score ranged from approximately 40 to 55 points at 3–12 months post-intervention. Pooled analysis using a random-effects mode demonstrated a large and clinically meaningful reduction in NOSE scores, well exceeding the established minimal clinically important difference of 30 points.

Short- and mid-term outcomes were consistent across surgical techniques, with improvements generally sustained at follow-up beyond 6 months. Heterogeneity was moderate to high, reflecting variability in patient selection, surgical technique, and follow-up duration. Sensitivity analyses excluding studies at high risk of bias did not materially alter the direction or magnitude of effect.

### 3.4. Risk of Bias and Quality of Evidence

Overall risk of bias was moderate to high, driven primarily by the predominance of uncontrolled observational studies. Only one randomized controlled trial provided Level I evidence. Diagnostic studies frequently lacked a blinded reference standard and showed variability in patient selection.

According to GRADE criteria, the overall certainty of evidence was low for diagnostic accuracy and low to moderate for surgical treatment outcomes, despite the consistency of large observed effect sizes.

### 3.5. Surgical and Minimally Invasive Treatment Studies: A Critical Appraisal

Patient satisfaction with spreader grafts is generally high, although some may notice a slight broadening of the nasal dorsum. Complication rates specific to spreader grafts are low; the main issues can be graft visibility or edges if not carved smoothly, and very rarely graft resorption or displacement. In our review, spreader grafts were often done alongside septoplasty (to harvest cartilage) and turbinate reduction, making it hard to isolate their effect. Comparative evidence, though limited, suggests that adding spreader grafts to septoplasty yields better functional outcomes than septoplasty alone in patients with narrow valve angles. A small prospective cohort study (*n* = 17) by Erickson et al. noted that patients with septal deviation plus internal valve narrowing had superior improvement when septoplasty was combined with spreader grafts versus septoplasty alone [10]. While statistically significant improvements were demonstrated across all outcomes, the lack of a control group and the inability to isolate the contribution of spreader grafts from the concomitant septoplasty, combined with only short-term follow-up (maximum ~18 weeks) and a predominantly male population from a single tertiary center, limit the attribution of findings to spreader graft placement alone.

In our included studies, alar batten grafts were frequently used in combination with spreader grafts when both internal and external valves were problematic. Khosh et al. conducted a retrospective case series of 53 patients from a single institution over an 8-year period using alar batten grafts in approximately 36% of their cases, reporting that all patients with external valve dysfunction improved after surgery [24]. Specifically, 12 out of 12 patients with primarily external collapse were relieved of obstruction postoperatively [24]. Despite a minimum one-year follow-up, multiple confounding factors—including the absence of a control group, reliance on unvalidated subjective outcomes without objective assessment, a highly heterogeneous cohort undergoing concurrent surgical techniques for mixed pathology, a predominance of post-rhinoplasty cases, and inherent selection and recall bias from the retrospective design—preclude reliable attribution of outcomes to any specific intervention. Other studies similarly report >90% patient satisfaction with breathing after batten graft placement. Toriumi et al. followed 46 patients with external or internal nasal valve collapse who received batten grafts and found that 45 of them (98%) had significant subjective improvement [25]. The mean improvement in nasal airway obstruction was 2.5 on a scale of 5 in a mean postoperative follow-up of 5 years [25]. On the other hand, the findings of this retrospective case series, based on a non-randomised design with incomplete follow-up and reliance on subjective patient-reported outcomes, are subject to significant confounding from concomitant nasal procedures and patient selection, and are further limited by lack of a control group. Batten grafts carry some aesthetic considerations—Khosh’s study noted that batten grafts resulted in “effacement of deep alar creases and a widening of the nasal tip” in some cases [24]. Patients should be counseled about these possible cosmetic changes, which can actually be desirable if the patient has pinching or inverted alar contours, but might be a trade-off for others. Complications specific to batten grafts are uncommon; warping of the cartilage is possible, and in thick-skinned noses the added volume might be noticeable as fullness. No study in our review showed any significant rate of graft infection or extrusion for batten grafts. When it comes to comparing spreader vs. batten graft outcomes, there have been few direct comparisons because they address different anatomic targets. However, a systematic review by Rhee et al. of 25 years of English-language literature aggregated various functional rhinoplasty techniques, including both spreaders and battens, and found no major differences in overall success rates between techniques—success was more dependent on using the right tool for the right patient [26]. High reported improvement rates (≈80–90%) across techniques suggest that both spreader and batten grafts may be effective when appropriately indicated, though evidence quality is limited by the low quality of included studies—42 of 44 were level 4 case series, with no randomized trials—and marked heterogeneity precluded meta-analysis.

Cartilage grafts and maneuvers intended to support the alar rim and the lower lateral cartilage framework improve the external valve and may alter the nasal tip position to a more open configuration. Gunter and Friedman (1997) presented an uncontrolled retrospective case series of 118 patients from a single institution describing the use of lateral crural strut grafts to prevent or correct medial movement of the lateral crus upon transdomal suturing for boxy nasal tips [27]. In our review, outcomes specific to these grafts were less frequently reported than for spreader or batten grafts, likely reflecting their use within broader rhinoplasty procedures and associated confounding. The retrospective case series by Di Stadio and Macro evaluated an “autologous alar cartilage grafting” technique for pinched nasal tip [28]. They found an 87.5% patient satisfaction rate and objective rhinomanometry improvement in all cases (25–75% airflow increase as mentioned), suggesting that directly addressing the alar rim and lateral crura is effective for external valve collapse in cases of collapsed nostril margins. Precaution should be taken with this interpretation, however, as only 12 patients were studied, a non-validated self-survey was used, and there is no statistical comparison between pre- and post-operative rhinomanometry values beyond descriptive percentage ranges. Another observational case series of 35 patients, the majority of whom underwent columelloplasty in combination with other nasal procedures, demonstrated improved breathing and external valve function with essentially no negative cosmetic trade-off [29]. It was primarily a technique report with 6-month–3-year follow-up, lacking validated or objective outcomes, controls, or independent assessment; 94% underwent concurrent procedures, making it a level 5 expert opinion with no reliable evidence for columelloplasty’s functional efficacy. Nevertheless, it seems that structural augmentation of the ala and tip should be considered when external collapse is present. It is also worth noting that although a standard technique may be used, cartilage grafting is typically individualized to the patient’s specific deformity, further increasing potential confounding. Patients in the 44-patient retrospective study of Vaezeafshar et al. undergoing repair of lateral wall insufficiency with alar rim grafts improved from mean NOSE 70 to 25 [30]. However, concurrent procedures, single-surgeon cohort, no untreated controls, and subjective grading limit data interpretability. No study demonstrated the superiority of any particular external technique; choice often reflects surgeon preference.

The suspension suture technique, which mimics the effect of the Cottle maneuver, is a less popular method of addressing valve collapse. André and Vuyk in 2008 reported mixed outcomes using this technique: about 21% no change, 52% slight improvement, and 27% marked improvement, with an average of 2.3/10 VAS improvement [31]. They also noted 9 of 33 nasal sides had complications such as suture extrusion, pain and loss of tension [31]. Although a prospective study, only 20 patients were included and the mean follow-up was 5 months. Moreover, only 5 patients had suspension alone, who showed a mean improvement of only 1 point. Schlosser and Park conducted a retrospective clinical case series of 34 patients, reporting that flaring sutures combined with spreader grafts yield significant improvement [32]. They did not, however, use the validated NOSE scale, they had a 12% loss to follow-up and they had a predominantly post-surgical and post-traumatic revision population (71%). In our review, no modern study was found that uses sutures alone as the sole method, suggesting that alar suspension sutures play a minor role in NVD treatment, showing lower efficacy on their own and risk needing revision [32,33,34,35].

Inferior turbinate reduction, though not a direct valve procedure, is frequently performed alongside valve surgeries to address any mucosal contribution to nasal obstruction. The inferior turbinate, particularly the anterior end, forms the floor of the internal nasal valve and if hypertrophied can significantly narrow the valve area [6]. In Sidle’s 2020 prospective, multicenter, nonrandomized single-arm study, 61 of 166 patients underwent turbinate reduction along with implant placement, and their outcomes were similar to those without turbinate work, implying turbinate reduction did not harm results and likely helped some patients [18]. The predominantly White cohort (86%) limits generalizability, and follow-up ended at 12 months despite the implant’s absorption over 18–24 months, collectively constraining conclusions regarding the device’s independent and long-term efficacy. Khosh et al. noted that in their series, whether or not turbinate reduction was done did not affect the high success rate of valve reconstruction [24]. The 2010 consensus recommended that septoplasty and/or turbinate surgery should be done when indicated in NVD—indeed sometimes a deviated septum or enlarged turbinate is the actual cause of “valve” compromise [3]. Our review did not find any cases where turbinate reduction worsened outcomes; if anything, it improves airflow and can enhance the results of valve repair.

## 4. Discussion

This systematic review provides a comprehensive overview of both diagnostic methods and surgical treatments for nasal valve dysfunction over the past 30+ years. Diagnosing NVD requires a nuanced approach combining symptom assessment, physical exam, and judicious use of objective tests. Our findings reinforce that no single diagnostic modality can stand alone: history and exam remain paramount, but objective confirmation of nasal airflow limitation can enhance diagnostic confidence. Determining when a patient’s obstruction is truly due to valve dysfunction versus other factors can be challenging. Tools like rhinomanometry and acoustic rhinometry, though underutilized, provide measurable evidence of valve compromise [1,2,36,37]. Yet, these measures do not always correlate strongly with how the patient feels, which is crucial in a condition defined by subjective sensation of airflow. This paradox—objective vs. subjective mismatch—is well-documented in nasal physiology [38,39]. One explanation lies in the concept of mucosal sensory feedback. Recent CFD studies indicate that the sensation of airflow is related to the cooling of nasal mucosa by airflow; even if the lumen is anatomically open, if airflow is not reaching certain receptors (e.g., due to flow rerouting from a deviation), a patient may feel obstructed [4,14,15,16]. Conversely, a slightly tight anatomy might be asymptomatic if airflow patterns still adequately stimulate nasal airflow receptors. Thus, comprehensive evaluation should pair an objective test with a subjective score.

Our review underscores the importance of the clinical exam in diagnosing NVD, while also advising against reliance on any single exam finding. In this context, the Cottle maneuver is not recommended as a standalone diagnostic tool due to its poor specificity and should instead be used as an adjunct within a comprehensive clinical assessment. The clinical exam should be systematic: inspect for inspiratory alar collapse externally, examine for septal deviation and turbinate hypertrophy, and use an endoscope to visualize the internal valve angle and collapse on inspiration. The modified Cottle or use of temporary dilators can be integrated into the exam to functionally test the valve and distinguish whether the obstruction is at the internal or external valve. This directly guides the surgical approach. The lack of consensus on diagnostic criteria for NVD, also noted by Pirola et al. [6], is an issue that this review highlights. Some surgeons diagnose NVD primarily on patient history (e.g., “better when I pull my cheek”), while others require visual confirmation of collapse, and others might use objective cut-offs, such as minimal area < 0.5 cm^2^ on acoustic rhinometry. This inconsistency can lead to variability in reporting outcomes and selecting patients for surgery. Moving forward, adoption of standardized diagnostic protocols would benefit research and practice. One potential approach is a diagnostic algorithm as proposed by Pirola et al., who suggest treating any septal deviation or turbinate hypertrophy first and addressing the valve specifically if obstruction persists [6]. While the algorithm avoids over-treating valve issues when simpler septal surgery might suffice, it is based on a single database search and non-systematic methodology. Moreover, in clinical practice, staged or multiple procedures are generally avoided.

**Figure 3:**
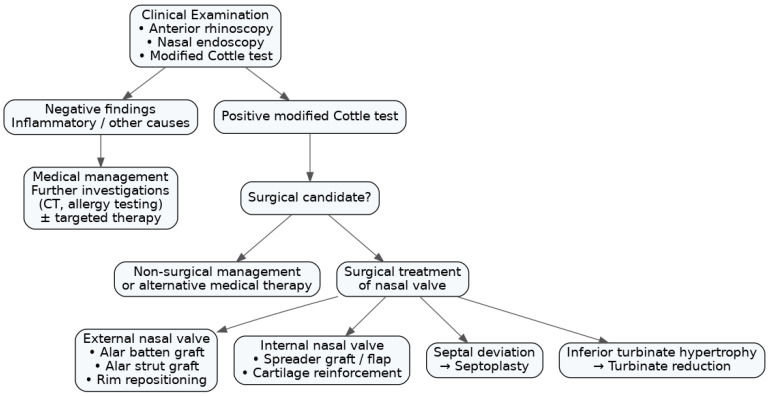
Diagnostic and treatment algorithm for nasal valve dysfunction. Clinical evaluation includes anterior rhinoscopy, nasal endoscopy, and the modified Cottle test. Patients with negative findings or features suggestive of inflammatory or alternative causes undergo further investigations and targeted medical management. A positive modified Cottle test prompts assessment of surgical candidacy. Patients unsuitable for surgery are managed conservatively, while surgical candidates undergo nasal valve surgery tailored to the level of obstruction, including treatment of the external nasal valve, internal nasal valve, septal deviation, and/or inferior turbinate hypertrophy.

Regarding surgical management, we found it yields tangible benefits, resolving many cases of chronic nasal obstruction that were unresponsive to medical therapy or septoplasty alone [32,33], with quality-of-life benefits [26,40]. The challenge is prospectively determining when intervention on the nasal valve is warranted. The retrospective data by Green et al. shows that only ~2% of septoplasty patients later needed valve surgery [41], suggesting that indeed careful patient selection for initial valve surgery is important. We propose a diagnostic and treatment algorithm designed to support clinical decision-making in addressing this issue (Figure 3).

Patient-reported outcomes are critical in functional nasal surgery, and the prevalent use of the NOSE scale in this review represents a methodological strength in the current literature. Functional nasal valve procedures inevitably modify nasal anatomy, making aesthetic outcomes another important consideration. Modern techniques and materials, using autologous cartilage and meticulous suturing, have likely improved this aspect. Further research could also explore other quality of life parameters that may be influenced by nasal valve intervention, such as risk of chronic rhinosinusitis or exercise tolerance.

Comparative effectiveness research is also needed. Future studies should ideally compare traditional cartilage graft surgery with minimally invasive alternatives in randomized trials. Controlled comparisons of different graft techniques—for example, spreader graft versus flaring suture versus absorbable implant—could clarify outcomes and cost-effectiveness, helping optimize patient care. Advancement and refinement of objective diagnostic measures also remain essential. For example, the Forced Inspiratory Effort Dynamics (FRIED) test, which reportedly identifies valve collapse with ~82% sensitivity [22], and rapid office-based stress tests could, if validated, enhance and standardize nasal valve assessment. This review also underscores the importance of long-term follow-up. Recent data include two-year follow-up for implants, which show maintained improvement, and four-year outcomes from a radiofrequency extension study, demonstrating continued benefit in a substantial proportion of patients [42]. However, obstruction may recur with tissue changes or aging, sutures can loosen and radiofrequency relies on scarring, highlighting the need for 5–10-year or longer follow-up studies.

The predominance of Level IV evidence represents the main limitation of our study. While the magnitude of the observed effect sizes is large and consistent reporting of positive outcomes provides some support for effectiveness, the certainty of evidence for treatment data remains low to moderate. Publication bias, particularly the tendency for surgeons to report favorable results, likely contributes to this. Moreover, pooling outcomes across heterogeneous designs limits the validity of quantitative synthesis. The observed heterogeneity likely reflects substantial variability in surgical techniques, patient selection, and follow-up duration across included studies. Although subgroup and meta-regression analyses would have been valuable to explore sources of heterogeneity, these were not feasible due to the limited number of studies with sufficiently homogeneous reporting of key variables. Another limitation is the inability to isolate the independent effect of valve interventions or, more specifically, the effect of interventions targeting the lateral nasal wall cartilage. As the valve is a functional cross-sectional area bounded by multiple structures, including the middle turbinate laterally and the septum medially, many patients underwent concomitant procedures, such as septoplasty or turbinate reduction as part of their treatment. Future studies should account for concomitant procedures to better clarify the independent effect of interventions targeting specific components of the nasal valve. Furthermore, several included studies did not specify their criteria for defining dysfunction of the nasal valve, which challenges the interpretation of pooled outcomes.

This review provides several practical considerations for clinicians: (1) Employ a validated symptom instrument, such as the NOSE scale, for all patients with nasal obstruction to quantify baseline severity and monitor outcomes. (2) When nasal valve dysfunction is suspected, consider objective measures (e.g., PNIF) to document impairment and response to diagnostic maneuvers, which can also support justification for intervention. (3) Surgical planning should address all levels of obstruction—valve, septum, and turbinates—to optimize outcomes, rather than employing a piecemeal approach. (4) For patients averse to formal rhinoplasty, emerging minimally invasive techniques (radiofrequency or absorbable implants) may be considered, recognizing that long-term efficacy remains under evaluation, though short-term results are encouraging. (5) Cosmetic implications should be discussed with patients, emphasizing that functional improvement is the priority, while subtle changes in nasal appearance may occur.

Our meta-analysis demonstrated mean NOSE score improvements of approximately 45 points, sustained at 12 months. These results can inform patient counseling regarding expected symptomatic relief. On average, patients experience a reduction in perceived nasal obstruction of roughly 50% on a 100-point scale, with 85–90% achieving a clinically meaningful improvement [24,26]. These findings provide a quantitative basis for setting realistic yet optimistic expectations for functional outcomes.

## 5. Conclusions

Nasal valve dysfunction is a significant cause of chronic nasal obstruction that can impair quality of life. This systematic review demonstrates that a wide array of diagnostic tools are available to evaluate NVD, each contributing unique information. Combining subjective patient reports with objective airflow measurements and thorough anatomical examination yields the best diagnostic accuracy. Clinicians should be aware of the limitations of any single test and have a complementary approach for diagnosing NVD.

A range of surgical techniques—from cartilage grafts to newer minimally invasive procedures—have been described for the management of nasal valve dysfunction. Available literature, primarily observational, generally reports symptomatic improvement with these interventions when appropriately applied. Traditional approaches such as spreader grafts appear effective for internal valve narrowing, while alar batten and rim grafts can address external valve collapse, frequently yielding high rates of patient-reported improvement. Due to clinical and methodological heterogeneity, our meta-analysis demonstrates that various treatment approaches can each improve nasal breathing (mean NOSE reduction ~45–50 points), but it does not allow direct comparison of their relative effectiveness. No single technique can be considered superior; selection should be tailored to the patient’s anatomic characteristics, often combining approaches to optimize outcomes. Rigorous RCTs and comparative studies are required to determine intervention efficacy, long-term outcomes, and subgroup-specific effects, with control of confounding variables.

Novel therapies, including absorbable lateral wall implants and in-office radiofrequency treatment, have broadened options for managing lateral wall insufficiency. Early Level I evidence suggests radiofrequency is safe and effective, with short-term outcomes comparable to traditional surgery, although long-term comparative data remain limited. These minimally invasive approaches may be particularly suitable for patients who are poor surgical candidates or who wish to avoid formal rhinoplasty.

Patients with nasal valve dysfunction can be counseled that effective interventions exist, with most achieving substantial improvements in nasal breathing supported by both subjective and objective measures. Our proposed diagnostic and treatment algorithm may help clarify surgical indications, while ongoing innovations continue to enhance functional outcomes and quality of life.

## Figures and Tables

**Table 1:** Eligibility criteria for study inclusion and exclusion.

Inclusion Criteria	
**Population**	Human subjects with nasal airway obstruction attributed to internal nasal valve dysfunction, external nasal valve dysfunction, or combined nasal valve pathology.
**Study design**	Randomized controlled trials (RCTs), non-randomized comparative studies, prospective or retrospective cohort studies, and case series including ≥10 patients.
**Interventions–Diagnostic**	Diagnostic modalities for nasal valve dysfunction, including clinical maneuvers, nasal endoscopy, peak nasal inspiratory flow (PNIF/NPIF), rhinomanometry, acoustic rhinometry, imaging-based assessments (CT/MRI), or computational fluid dynamics (CFD).
**Interventions–Therapeutic**	Surgical or minimally invasive treatments targeting nasal valve dysfunction, including spreader grafts, alar batten grafts, lateral crural strut or rim grafts, suture suspension techniques, bioabsorbable implants, and temperature-controlled radiofrequency remodeling.
**Outcomes–Diagnostic**	Studies reporting sensitivity and specificity of diagnostic methods.
**Outcomes–Therapeutic**	Studies reporting at least one clinically relevant outcome, including validated symptom scores (e.g., NOSE, VAS), objective airflow measurements, or postoperative functional improvement.
**Publication** **characteristics**	Articles published in English between January 1990 and January 2026.
**Exclusion Criteria**	
**Small sample size**	Case reports and case series including <10 patients.
**Non-clinical studies**	Cadaveric or purely anatomical studies without clinical outcome data.
**Population**	Studies involving pediatric-only populations.
**Publication type**	Narrative reviews, expert opinions, editorials, and conference abstracts without full-text data.
**Intervention** **relevance**	Studies in which nasal valve interventions could not be clearly distinguished from other nasal or sinonasal procedures.

**Table 2:** Summary of diagnostic tools for nasal valve dysfunction. Performance values are given where available.

Diagnostic Tool	Sensitivity	Specificity	Comments
**Cottle test** (lateral cheek pull)	Very high (~97%+)	Very low (~0%)	Almost all individuals show some airflow improvement with this maneuver, so a positive test lacks specificity for NVD (many false-positives in normals) [5,6].
**Modified Cottle maneuver**	–	–	Performed by selectively supporting internal vs. external valve areas. Can help localize the level of collapse (internal vs. external) and predict which surgical approach is needed. Strong correlation with surgical outcomes when used appropriately [7].
**Rhinomanometry** (4-phase)	~88%	~90%	Objective measurement of nasal airway resistance. Four-phase rhinomanometry accurately detects internal valve collapse [1]. Requires specialized setup; active (patient-driven) rhinomanometry is effort-dependent.
**Acoustic rhinometry**	–	–	Uses sound waves to measure cross-sectional area/volume of nasal passages. Identifies anatomical narrowings (e.g., small MCA at valve) but provides a static snapshot. Poor correlation with symptom severity [8]. Mainly anterior measurements are reliable.
**Peak Nasal** **Inspiratory Flow** (PNIF)	~75%	~70%	A simple handheld peak flow meter to measure maximal nasal inspiratory flow. Effort-dependent and needs patient cooperation. A <20 L/min (or <20%) improvement after decongestant indicates likely structural obstruction (fixed valve issue) [9]. Normative values vary by age and sex.
**Nasal endoscopy**	–	–	Direct visualization of internal anatomy and dynamic motion. Allows identification of structural causes (septum, turbinate, valve area collapse) and grading of collapse. Moderate interobserver agreement on collapse severity [10]. Complements external exam; can detect concurrent pathologies (polyps, etc.).
**VAS** **(Visual Analog Scale)**	–	–	Patient-rated severity of nasal obstruction (e.g., 0–10 or 0–100 scale). Quick and easy but entirely subjective. Often used to track change pre- vs. post-treatment rather than to diagnose the cause.
**NOSE questionnaire**	–	–	A validated 5-item patient survey for nasal obstruction (score 0–100). Useful for quantifying impact and outcomes; MCID ~30 points [11,12]. Not a diagnostic tool by itself, but essential for patient-reported outcome measurement.
**Imaging (CT/MRI)**	–	–	Provides detailed anatomy of nasal structures and can measure valve angles or area. Helpful for surgical planning (e.g., identifying high septal deviations, assessing turbinate bone size). Static images do not show dynamic collapse; studies show limited correlation with functional improvement [13]. Radiation (CT) or cost (MRI) considerations limit routine use.
**Computational Fluid Dynamics** (CFD)	–	–	Advanced simulation of nasal airflow using patient-specific anatomy. Research studies demonstrate correlation of CFD metrics (airflow distribution, mucosal cooling) with patient-reported patency [4,14,15,16]. Not yet clinically practical for routine diagnosis, but holds promise for understanding complex cases and surgical planning.

**Table 3:** Characteristics of the 72 included studies.

Author(s), Year [Ref]	Study Design	Intervention/Diagnostic Modality	Primary Outcome	Key Results	Follow-Up
**Gagnieur p et al., 2022** [1]	Prospective diagnostic accuracy	Four-phase rhinomanometry for INVC diagnosis	Sensitivity/specificity for INVC	Four-phase rhinomanometry: sensitivity ~88%, specificity ~90% for INVC. Highest diagnostic performance among objective tests.	NR
**Das a, spiegel jh, 2020** [5]	Prospective diagnostic study	Cottle maneuver validity and specificity in NVC	False-positive rate in healthy individuals	98% of healthy individuals reported improved airflow with Cottle maneuver; extremely high false-positive rate, limiting standalone diagnostic value	Single encounter
**Fung e et al., 2014** [7]	Prospective cohort	Modified Cottle maneuver to predict functional rhinoplasty outcomes	Correlation of pre-op modified Cottle with post-op NOSE	Modified Cottle correctly identified INVD vs. ENVD site; strong correlation with post-operative outcomes	Post-op follow-up
**Toyserkani nm et al., 2013** [8]	Prospective long-term cohort	Acoustic rhinometry post-septoplasty	Correlation with NOSE (pre-op, 3 mo, 11-yr)	No significant correlation between acoustic rhinometry and NOSE scores at any time point; highlights objective–subjective mismatch	11 years
**Chin d et al., 2014** [9]	Cross-sectional diagnostic study	NPIF to differentiate structural vs. decongestable obstruction	Sensitivity/specificity for structural NVD	PNIF < 60 L/min normalising to >120 L/min with dilator strongly suggests surgically correctable NVD; effort-dependent, limiting specificity	Single encounter
**Erickson b et al., 2016** [10]	Prospective cohort	Endonasal spreader graft + septoplasty + turbinoplasty for NVC	Acoustic rhinometry, video endoscopic scoring, NOSE, SNOT-22	Significant improvement in endoscopic collapse grade, MCA, NOSE, and SNOT-22; septoplasty + spreader grafts superior to septoplasty alone in narrow valve angles	8–18 weeks
**Rhee js et al., 2014** [11]	Systematic review of PROMs	NOSE scale normative/symptomatic range definition	Normative and symptomatic NOSE score ranges	NOSE ≥ 55 = severe/extreme obstruction; MCID = 30 points; foundational reference for outcome interpretation in functional nasal surgery	Various
**Stewart mg et al., 2004** [12]	Development and validation study	Development and validation of NOSE scale	Reliability, construct validity of NOSE scale	NOSE scale validated as reliable, valid disease-specific outcome measure for nasal airway obstruction; 5-item, 0–100 scale	NR
**Menger dj et al., 2014** [13]	Retrospective case series	Surgery of external nasal valve—subjective/objective correlation	ROE score vs. rhinomanometry	Moderate correlation between subjective (ROE) and objective (rhinomanometry) measures; ROE not NVD-specific	NR
**Sullivan cd et al., 2014** [14]	Prospective CFD/clinical study	CFD: mucosal cooling and nasal patency post-surgery	Nasal patency perception vs. CFD mucosal cooling	Increased mucosal cooling correlates with better post-surgical nasal patency perception; mechanistic insight into NVD physiology	Post-op
**Zhao k et al., 2014** [15]	Prospective CFD study	CFD: regional peak mucosal cooling and nasal patency perception	Correlation of CFD mucosal cooling with subjective patency	Regional peak mucosal cooling predicts subjective nasal patency; CFD metric correlates with patient-reported symptoms	NR
**Casey kp et al., 2017** [16]	Prospective CFD/clinical study	CFD: intranasal airflow distribution and subjective patency	Correlation of airflow distribution with NOSE score	Intranasal airflow distribution correlates moderately with NOSE scores; supports CFD as future diagnostic adjunct	NR
**Wang y, bonaparte jp, 2019** [21]	Cross-sectional survey	Survey on NVD diagnostic and management practices	Practice pattern heterogeneity	63% use Cottle test for external valve; <40% use objective tests; major heterogeneity; underuse of validated diagnostic tools	Single survey
**Maalouf r et al., 2016** [22]	Prospective diagnostic accuracy	FRIED test to differentiate NVC from other nasal obstruction	Sensitivity of FRIED test for NVC	FRIED test sensitivity ~82% for NVC; potential rapid office-based diagnostic tool; requires further validation	Single encounter
**Wu j et al., 2024** [23]	Retrospective CT study	CT-derived MCA vs. acoustic rhinometry vs. NOSE correlation	Correlation of CT imaging with NOSE and acoustic rhinometry	CT-derived MCA correlated better with NOSE scores than acoustic rhinometry MCA; imaging as useful anatomical adjunct	NR
**Floyd em et al., 2017** [17]	Secondary systematic review	Functional rhinoplasty (various NVD techniques)—25-yr literature	NOSE score improvement across techniques	Success rates ≈ 80–90%; no technique superior; 42/44 studies Level IV; heterogeneity precluded meta-analysis; NOSE mean improvement ~40–50 pts	Variable
**Sidle dm et al., 2020** [18]	Prospective multicenter non-randomised	Bioabsorbable lateral wall implant (LATERA) ± turbinate reduction	NOSE score at 12 months	Significant NOSE improvement at 12 months; concomitant turbinate reduction had similar outcomes; 86% White cohort; follow-up limited to 12 months	12 months
**Han jk et al., 2022** [19]	Prospective non-randomised (open-label)	Temperature-controlled RF (Vivaer) for NVC—12-month outcomes	NOSE score at 12 months	Significant NOSE improvement at 12 months; consistent with RCT active arm; multicenter, real-world setting	12 months
**Silvers sl et al., 2021** [20]	Randomised Controlled Trial	Temperature-controlled RF device (Vivaer) vs. sham for NVC	NOSE score (primary: 3 mo; secondary: 12 mo)	Active arm: significant NOSE reduction vs. sham at 3 months; 14% crossover; ITT analysis; only Level I evidence in review	12 months
**Khosh mm et al., 2004** [24]	Retrospective case series	Nasal valve reconstruction (spreader + batten grafts, multi-technique)	Subjective breathing improvement (non-validated scale)	12/12 ENVD patients relieved postoperatively; all patients improved; turbinate reduction did not affect outcome; single institution; post-rhinoplasty predominance	≥12 months
**Toriumi dm et al., 1997** [25]	Retrospective case series	Alar batten grafts for internal and external NVC	Subjective 5-point airway obstruction scale	45/46 (98%) significant subjective improvement; mean improvement 2.5/5 on airway scale; non-validated outcome measure	Mean 5 years
**Rhee js et al., 2008** [26]	Secondary systematic review	Functional rhinoplasty/NVD repair—25-year evidence review	Overall success rates across all techniques	Success rates ≈ 80–90%; no major differences between spreader vs. batten grafts; evidence quality limited (42/44 Level IV)	Variable
**Gunter jp, friedman rm, 1997** [27]	Retrospective case series	Lateral crural strut grafts for nasal tip/external valve correction	Subjective patient-reported airway improvement	Technique description with large series; externally validated widely; non-validated outcome measure; LCSG-specific effect difficult to isolate	NR
**Di stadio a, macro c, 2018** [28]	Retrospective case series	Flip-flap alar technique for pinched nasal tip/external valve collapse	Patient satisfaction + rhinomanometry	87.5% satisfaction; 25–75% airflow increase on rhinomanometry; limited by small *n* = 12, non-validated survey, no statistical comparisons	NR
**Fanous n, 1990** [29]	Retrospective case series	Columelloplasty for wide obstructing columella/external valve	Subjective breathing + cosmetic outcome	Improved breathing and external valve function; 94% concurrent procedures; no validated outcomes; Level V evidence	6 months–3 years
**Vaezeafshar r et al., 2018** [30]	Retrospective case series	Alar rim grafts for lateral wall insufficiency/external valve	NOSE score (pre- and post-op)	Mean NOSE improved from 70 to 25; concurrent procedures; single-surgeon; no control group	NR
**André rf, vuyk hd, 2008** [31]	Prospective case series	Valve suspension suture technique for NVC	VAS nasal obstruction (0–10)	21% no change, 52% slight improvement, 27% marked improvement; mean VAS improvement 2.3/10; 9/33 nasal sides had complications (extrusion, pain)	Mean 5 months
**Schlosser rj, park ss, 1999** [32]	Retrospective case series	Flaring sutures + spreader grafts for dysfunctional nasal valve	Subjective non-validated scale + nasal patency	Significant improvement reported; non-validated scale; 12% lost to follow-up; 71% post-surgical or traumatic revision population	NR
**Paniello rc, 1996** [33]	Retrospective case series	Nasal valve suspension sutures—original technique description	Subjective 5-point patency score	Original suture suspension technique description; foundational reference for non-graft NVD repair	NR
**André rf, vuyk hd, 2008** [34]	Technique note/small series	Butterfly graft for internal nasal valve incompetence	NVD functional outcomes	Technique description; cited for comparative context on butterfly graft for INVD	NR
**O’halloran lr, 2003** [35]	Retrospective case series	Lateral crural J-flap repair for NVC	Subjective airway improvement	Technique description; cited as comparator for suture/flap-based NVD techniques	NR
**Vogt k, zhang l, 2012** [36]	Methodology review	Four-phase rhinomanometry methodology in septal surgery	Rhinomanometry utility and technique	4-phase rhinomanometry most reliable objective test for nasal airway; provides normative reference values for NVD assessment	N/A
**Vogt k et al., 2016** [37]	Retrospective multicentre methodology	Four-phase rhinomanometry—multicentric normative analysis	Normative data validity across multiple centres	Large normative dataset confirming 4-phase rhinomanometry reliability; reference values for NVD diagnostic context	NR
**Edizer dt et al., 2013** [38]	Prospective case series (septorhinoplasty)	Acoustic rhinometry post-septorhinoplasty	Acoustic rhinometry vs. VAS correlation	Acoustic rhinometry less reliable post-rhinoplasty; variable VAS correlation; objective–subjective mismatch in mixed population	Post-op
**Haavisto le, sipilä ji, 2013** [39]	Prospective 10-year follow-up (septoplasty)	Acoustic rhinometry, rhinomanometry, VAS post-septoplasty	Long-term objective–subjective correlation	Objective–subjective mismatch persists at 10 years; highlights limitations of objective measures as sole endpoints	10 years
**Zainea v, 2001** [40]	Textbook/reference monograph	NVD pathology and surgery (comprehensive reference)	Classification and surgical principles for NVD	Foundational Romanian-language monograph on NVD pathology and surgical approach; cited for classification and historical context	N/A
**Green a et al., 2024** [41]	Retrospective cohort	Septoplasty for nasal valve collapse—revision rate analysis	Proportion requiring valve surgery after septoplasty	Only ~2% of septoplasty patients subsequently required valve surgery; supports careful primary patient selection	NR
**Jacobowitz o et al., 2022** [42]	Prospective long-term follow-up	Temperature-controlled RF for NVC—4-year outcomes	NOSE score at 4-year follow-up	Continued symptomatic benefit at 4 years; longest reported follow-up for RF treatment in NVD	4 years
**Kimbell js et al., 2013** [4]	Prospective CFD/clinical study	CFD analysis of airflow and heat transfer post-nasal obstruction surgery	Correlation of airflow/heat transfer changes with symptom improvement	Changes in nasal airflow and heat transfer correlate with patient-reported symptom improvement; mechanistic insight into NVD physiology	Post-op
**Rhee js et al., 2010** [3]	Clinical consensus statement	Diagnosis and management of NVD—AAO-HNS consensus 2010	Practice recommendations for NVD diagnosis and treatment	CT rarely helpful for NVD; septoplasty/turbinate surgery recommended when indicated; foundational consensus document	N/A
**Rimmer j et al., 2019** [2]	European position paper	Diagnostic tools in rhinology—European position paper	Evidence grading of diagnostic tools for nasal obstruction	Acoustic rhinometry and rhinomanometry most reliable objective tests; Cottle test least specific; PNIF useful screening tool	N/A
**Pirola f et al., 2025** [6]	Narrative review with management algorithm	NVD comprehensive literature analysis with proposed management algorithm	Diagnostic and treatment pathway recommendations	Algorithm: treat septal/turbinate pathology first; then address valve; based on single database, non-systematic methodology	N/A
**Han jk et al., 2025** [43]	Prospective long-term cohort	Temperature-controlled RF for NVC—long-term outcomes extension	NOSE score at extended follow-up (>2 years)	Sustained NOSE improvement at extended follow-up; cited for long-term evidence on minimally invasive RF treatment	Long-term (>2 yr)
**Brehmer d et al., 2019** [44]	Prospective observational outcomes study	Single-session bilateral TCRF treatment of the internal nasal valve	NOSE score; Snore Outcomes Survey (SOS)	Significant improvement in NOSE at 3 months; SOS improved significantly at 30 days and 3 months (*p* < 0.001)	3 months
**Most sp, 2006** [45]	Prospective case series	Functional rhinoplasty (spreader grafts, alar rim grafts, suture techniques) for NVD	NOSE score pre- and post-op	Significant NOSE improvement; disease-specific QoL instrument validated for NVD surgical evaluation; foundational outcomes study	NR
**Standlee ag, hohman mh, 2017** [46]	Retrospective comparative cohort	Spreader grafting vs. non-spreader techniques for nasal obstruction	NOSE score change across groups	Spreader grafting significantly improved NOSE; direct comparison of NVD surgical techniques; supports graft-specific patient selection	NR
**Fuller jc et al., 2019** [47]	Prospective cohort	Functional septorhinoplasty with spreader graft placement for NVD	NOSE + FACE-Q Nose/Nostrils + Social Functioning	NOSE improved from 62.7 to 22.8 (*p* < 0.001); all FACE-Q scores improved significantly; cosmetic outcomes favourable; no aesthetic penalty from spreader grafts	Up to 12 months
**Yeung a et al., 2016** [48]	Retrospective cohort	Functional and aesthetic-functional rhinoplasty for NVC (INVD, ENVD)	NOSE score change from baseline	Significant NOSE improvement in both functional-only and combined groups; no significant difference between groups; supports combined approach	NR
**Barham hp et al., 2015** [49]	Retrospective comparative	Costal cartilage LCSG vs. cephalic crural turn-in for external valve dysfunction	NOSE score, PNIF, rhinomanometry	Both techniques improved NOSE and objective airflow; costal cartilage LCSG favoured for revision cases; no significant outcome difference	NR
**Sowder jc et al., 2017** [50]	Prospective case series	Functional rhinoplasty for NVD (spreader flaps)	NOSE score at 3 and 12 months	Significant NOSE improvement at 3 and 12 months; sustained improvement consistent with NVD literature range	12 months
**Kondo m et al., 2020** [51]	Retrospective comparative cohort	Lateral crural tensioning with articulated alar rim graft (LCT/AARG) vs. LCSG for NVC	NOSE, VAS, PNIF, total nasal airway resistance (rhinomanometry)	LCT/AARG: greater reduction in total nasal airway resistance; both techniques improved NOSE and VAS; LCT/AARG preferable in narrow noses	6 months
**Naguib mb et al., 2020** [52]	Prospective randomized controlled clinical trial	Spreader grafts vs. auto-spreader flaps for internal nasal valve dysfunction	NOSE score pre- and postoperatively at 3 and 6 months	Both techniques significantly improved NOSE at 6 months; spreader flaps: shorter operative time, less cartilage use; comparable outcomes	6 months
**Albergo l et al., 2020** [53]	Prospective case series	Spreader graft with endonasal approach for septal deviation + INVD	NOSE score, rhinomanometry pre- and post-op	Significant improvement in NOSE (*p* = 0.001) and rhinomanometry (*p* < 0.001); complication rate 6%; endonasal approach effective for INV-specific pathology	12 months
**Ismail a et al., 2018** [54]	Prospective case series	Spreader graft + lateral suture suspension for narrow INVD	NOSE score, CT valve angle, endoscopic INV angle	NOSE decreased from 69.8 to 20.65; INV angle increased significantly bilaterally; 91% maintained relief at 3 years	3 years
**Tastan e et al., 2011** [55]	Prospective controlled study	Novel H-graft internal nasal valve reconstruction technique	NOSE score, ROE score at 12 months	Significant improvement in NOSE and ROE at 12 months; technique enables greater ULC lateralisation than standard spreader graft	12 months
**Hassanpour se et al., 2023** [56]	Randomised double-blinded clinical trial	Spreader graft with vs. without suturing to upper lateral cartilage	NOSE score, nasal appearance assessment	Both techniques improved NOSE; modified technique (without suturing to ULC) provided comparable functional and superior aesthetic outcomes	NR
**Raposo a et al., 2022** [57]	Retrospective comparative	Spreader grafts vs. spreader grafts + alar batten graft vs. modified alar batten graft for NVD	Surgical success rate for NVD correction	Modified alar batten grafts achieved superior outcomes over spreader grafts alone and spreader grafts combined with classic alar batten grafts.	NR
**Weitzman re et al., 2021** [58]	Prospective cohort	Standard vs. extended spreader grafts in septorhinoplasty for NVD	NOSE, FACE-Q Nose/Nostrils/Social Functioning at 6 and 12 months	Both SSG and ESG significantly improved NOSE and FACE-Q; no significant difference between groups at 6 or 12 months	12 months
**Rhee js et al., 2005** [59]	Prospective observational	Nasal valve surgery for NVD—disease-specific QoL improvement	NOSE score pre- and post-op	Nasal valve surgery significantly improves disease-specific QoL as measured by NOSE; foundational evidence for functional rhinoplasty benefit	NR
**Jacobowitz o et al., 2019** [60]	Prospective single-arm multicentre	In-office bipolar RF treatment for dynamic/static INVD	NOSE score at 6 months	Mean NOSE improvement 55.9 points at 6 months; 92.3% responders; foundational prospective study for in-office RF treatment of NVD	6 months
**Ephrat m et al., 2021** [61]	Prospective long-term follow-up	Temperature-controlled RF for NVC—24-month outcomes	NOSE score at 24 months	NOSE improvement maintained at 24 months (mean 53.5 pts); 97.2% responders; supports durability of in-office RF treatment	24 months
**Yamasaki a et al., 2019** [62]	Prospective case series	Functional rhinoplasty for NVD—lateral crural techniques	NOSE score, objective airflow measures	Significant NOSE improvement; lateral crural techniques effective for combined INVD and ENVD	NR
**Abdelwahab m et al., 2021** [63]	Retrospective case series	NVD repair—lateral crural strut grafting for external valve	NOSE score at last follow-up	Significant NOSE improvement; LCSG effective for lateral wall insufficiency; single-institution retrospective	NR
**Hismi a et al., 2022** [64]	Prospective longitudinal cohort study	Functional septorhinoplasty for lateral wall insufficiency	NOSE and FACE-Q score pre- and post-op	Significant NOSE improvement; outcomes consistent with literature range of 40–55 points; FACE-Q nasal satisfaction scores also improved significantly	~3.5 years
**Patel b et al., 2018** [65]	Prospective case series (functional rhinoplasty)	Septorhinoplasty for external NVC—INV grading system validation and outcomes	INV grading score, NOSE, SNOT-22, VAS, PNIF	All outcomes improved significantly post-op; INV grading system validated (Cronbach α 0.936); PNIF correlated with unilateral VAS; follow-up 18.8 weeks	18 weeks
**Sahin ms, ozmen oa, 2016** [66]	Prospective case series	Modified triangular spreader graft for INV area enlargement	NOSE score, VAS at 3 months	No complications; significant NOSE and VAS improvement at 3 months; modified technique safe and effective for INV enlargement	3 months
**Barone m et al., 2019** [67]	Retrospective case series (spreader flaps)	Spreader flaps for severe septal deviation—functional and long-term outcomes	NOSE score, rhinomanometry, long-term satisfaction	Spreader flaps effective for midvault support; functional improvement sustained long-term; tissue-preserving, no separate graft harvest	Long-term
**Talmadge j et al., 2018** [68]	Retrospective comparative	Open vs. endonasal spreader graft placement for NVD—NOSE outcomes	NOSE score comparison between approaches	Both open and endonasal approaches improved NOSE; no significant functional difference; approach selection based on surgeon preference and anatomy	NR
**Cervelli v et al., 2009** [69]	Prospective case series	Alar batten cartilage graft for internal and external nasal valve collapse	Subjective breathing outcome + rhinomanometry	Significant improvement in nasal patency and airflow; alar batten graft effective for both INV and ENV collapse	NR
**Clark jm, cook ta, 2002** [70]	Retrospective case series	Butterfly graft for functional secondary rhinoplasty (INVD)	Subjective patient satisfaction + nasal patency	Butterfly graft effective for INVD in revision rhinoplasty; no graft resorption or infection; improves internal valve angle	NR
**Rizvi ss, gauthier mg, 2011** [71]	Retrospective 10-year survey	Suture lateral suspension technique for NVC—10-year outcomes	Subjective breathing improvement + complication rate	Simplified suture technique; sustained improvement over 10 years; lower complication rate than classic suspension	10 years
**Pritikin j et al., 2023** [72]	Prospective single-arm multicentre	Temperature-controlled RF of septal swell bodies for nasal airway obstruction	NOSE score	Significant NOSE improvement; RF effective for septal swell body hypertrophy contributing to NVD; extends RF indications beyond lateral wall	NR

The first 15 studies represent the diagnostic studies. The remainder represent the 57 treatment studies. Abbreviations: AARG = articulated alar rim graft; CFD = computational fluid dynamics; ENVD = external nasal valve dysfunction; ESG = extended spreader graft; INVC/INVD = internal nasal valve collapse/dysfunction; ITT = intention to treat; LCSG = lateral crural strut graft; LCT = lateral crural tensioning; MCA = minimal cross-sectional area; MCID = minimal clinically important difference; N/A = not applicable; NOSE = Nasal Obstruction Symptom Evaluation; NR = not reported; NVC = nasal valve collapse; NVD = nasal valve dysfunction; PNIF/NPIF = peak nasal inspiratory flow; QoL = quality of life; RCT = randomised controlled trial; RF = radiofrequency; ROE = rhinoplasty outcomes evaluation; SNOT = Sino-Nasal Outcome Tool; SSG = standard spreader graft; ULC = upper lateral cartilage; VAS = visual analogue scale.

## Data Availability

The standardised data extraction template, extracted datasets, and statistical analysis code (RevMan/Stata) are available from the corresponding author upon reasonable request. The PRISMA flow diagram and risk-of-bias summaries are provided as Supplementary Materials.

## Supplementary Materials

The following supporting information can be downloaded at: https://www.mdpi.com/article/10.3390/diagnostics16091324/s1. Table S1: PRISMA 2020 Checklist; Table S2: PRISMA 2020 for Abstracts Checklist; Table S3: Studies Assessed for Eligibility at Full-Text Stage and Excluded, with Reasons; Figure S1: Risk of Bias Summary.
