# Supplementary material for: Diagnostic Approaches and Surgical Outcomes in Nasal Valve Dysfunction: A Systematic Review
Source: Diagnostics (Basel). 2026 Apr 28;16(9):1324. doi: 10.3390/diagnostics16091324 (PMC13164084; doi:10.3390/diagnostics16091324)
Supplement: Supplementary file 1 [file diagnostics-16-01324-s001.zip › Supplementary Table S3 corectat.pdf]

Supplementary Table S3

Studies Assessed for Eligibility at Full-Text Stage and Excluded, with Reasons

	Author(s) & Year	Full Reference (abbreviated)	Reason for Exclusion	Study Design	DB
**Category A — Wrong study design: narrative reviews, expert opinions, editorials, consensus statements, secondary systematic reviews / meta-analyses (PRISMA exclusion criterion: Publication type)**					
1	Wexler DB, Davidson TM (2004)	The nasal valve: a review of the anatomy, imaging, and physiology. Am J Rhinol 18(3):143–150	Narrative review; no primary patient outcome data	Narrative review	PubMed
2	Bloching MB (2007)	Disorders of the nasal valve area. GMS Curr Top Otorhinolaryngol Head Neck Surg 6:Doc07	Narrative review; descriptive overview, no original clinical data	Narrative review	PubMed
3	Most SP (2008)	Trends in functional rhinoplasty. Arch Facial Plast Surg 10(6):410–413	Expert opinion/editorial commentary; no primary outcome data	Editorial	PubMed
4	Hamilton GS 3rd (2017)	The external nasal valve. Facial Plast Surg Clin North Am 25(2):179–194	Narrative review chapter; no original clinical data	Narrative review	PubMed
5	Fraioli RE et al. (2013)	A patient with nasal valve compromise. JAMA Otolaryngol Head Neck Surg 139(9):947–950	Clinical vignette/educational case discussion; not a primary clinical study	Clinical vignette	PubMed
6	Lee DY et al. (2024)	Management of nasal valve dysfunction. Clin Exp Otorhinolaryngol 17(3):189–197	Narrative review; no original primary outcome data	Narrative review	PubMed
7	Spielmann PM, White PS, Hussain SS (2009)	Surgical techniques for the treatment of nasal valve collapse: a systematic review. Laryngoscope 119(7):1281–1290	Secondary systematic review; not a primary clinical study	Systematic review	PubMed
8	Sanan A, Most SP (2019)	A bioabsorbable lateral nasal wall stent for dynamic nasal valve collapse: a review. Facial Plast Surg Clin North Am 27(3):367–371	Narrative device review; no original clinical data	Narrative review	PubMed
9	Schuman TA, Senior BA (2018)	Treatment paradigm for nasal airway obstruction. Otolaryngol Clin North Am 51(5):873–882	Narrative review/treatment algorithm overview; no primary data	Narrative review	PubMed
10	Kim DH et al. (2020)	Effectiveness of using a bioabsorbable implant (Latera) to treat nasal valve collapse: systematic review and meta-analysis. Int Forum Allergy Rhinol 10(6):719–725	Secondary systematic review/meta-analysis; not a primary clinical study	Systematic review	PubMed
11	Kim DH et al. (2023)	Effectiveness of radiofrequency device treatment for nasal valve collapse: systematic review and meta-analysis. Am J Rhinol Allergy 37(5):636–644	Secondary systematic review/meta-analysis; not a primary clinical study	Systematic review	PubMed
12	Fearington FW et al. (2024)	Long-term outcomes of septoplasty with or without turbinoplasty: a systematic review. Laryngoscope 134(6):2525–2537	Secondary systematic review; septoplasty-only population, NVD not primary diagnosis	Systematic review	PubMed
**Category B — Non-clinical studies: cadaveric, anatomical, or primarily computational studies without primary clinical patient outcome data (PRISMA exclusion criterion: Non-clinical studies)**					
13	Schlosser RJ, Park SS (1999)	Surgery for the dysfunctional nasal valve: cadaveric analysis and clinical outcomes. Arch Facial Plast Surg 1(2):105–110	Primary component is cadaveric analysis (6 cadaver heads); clinical case series uses non-validated 5-point patency score; criterion E also applies	Cadaveric study + retrospective case series	PubMed
14	Bae JH, Most SP (2012)	Cadaveric analysis of nasal valve suspension. Allergy Rhinol (Providence) 3(2):e91–93	Pure cadaveric study; no clinical patient outcomes	Cadaveric study	PubMed
15	Coan BS et al. (2009)	Validation of a cadaveric model for comprehensive physiologic and anatomic evaluation of rhinoplastic techniques. Plast Reconstr Surg 124(6):2107–2117	Cadaveric model validation study; no clinical patient outcomes	Cadaveric study	PubMed
16	Wu Z et al. (2021)	Regional peak mucosal cooling predicts radiofrequency treatment outcomes of nasal valve obstruction. Laryngoscope 131(6):E1760–E1769	CFD modelling study (primary objective); NVD clinical outcome secondary to airflow modelling; reference standard insufficient for inclusion as primary diagnostic or treatment study	Prospective CFD + case series (n=20)	PubMed
17	Tarabichi M, Fanous N (1993)	Finite element analysis of airflow in the nasal valve. Arch Otolaryngol Head Neck Surg 119(6):638–642	Computational/mathematical modelling study; no clinical patient data	Computational study	PubMed
**Category C — Pediatric-only population (all patients < 18 years) (PRISMA exclusion criterion: Population)**					
18	Fuller JC et al. (2014)	Pediatric nasal valve surgery: short-term outcomes and complications. Int J Pediatr Otorhinolaryngol 78(8):1401–1404	Exclusively pediatric population (age 6–15 years); excluded per predefined criterion	Retrospective case series	PubMed
19	Moubayed SP et al. (2018)	External nasal valve repair in children: alar batten graft reinforced by external temporary suspensory suture. Ann Otol Rhinol Laryngol 127(10):729–733	Exclusively pediatric population (age 8–12 years); excluded per predefined criterion	Prospective case series	PubMed
**Category D — Insufficient sample size: case reports or series with < 10 patients, or technique reports without extractable NVD outcome data (PRISMA exclusion criterion: Small sample size)**					

	Author(s) & Year	Full Reference (abbreviated)	Reason for Exclusion	Study Design	DB
20	André RF, Paun SH, Vuyk HD (2004)	Endonasal spreader graft placement as treatment for internal nasal valve insufficiency without dividing the upper lateral cartilages from the septum. Arch Facial Plast Surg 6(1):36–40	Case series of 7 patients; below the predefined threshold of ≥10 patients	Case series (n=7)	PubMed
21	Ozturan O et al. (2002)	Bending of the upper lateral cartilages for nasal valve collapse. Arch Facial Plast Surg 4(4):258–261	Small case series below ≥10 patient threshold; primarily a technique description	Technique report/case series (<10)	PubMed
22	Park SS (1998)	The flaring suture to augment the repair of the dysfunctional nasal valve. Plast Reconstr Surg 101(4):1120–1122	Technique description with illustrative series of <10 patients with extractable NVD outcome data	Technique report (<10 patients)	PubMed
23	André RF, Vuyk HD (2008)	The 'butterfly graft' as a treatment for internal nasal valve incompetence. Plast Reconstr Surg 122(2):73e–74e	Letter/technique note; <10 patients with isolable butterfly graft NVD outcome data	Technique note (<10 patients)	PubMed
24	Paniello RC (1996)	Nasal valve suspension: an effective treatment for nasal valve collapse. Arch Otolaryngol Head Neck Surg 122(12):1342–1346	Original suture suspension technique description; small series with non-validated 5-point patency score and <10 patients for isolated suspension subgroup	Retrospective case series (<10 suspension-only)	PubMed
25	Nyte CP (2007)	Hyaluronic acid spreader-graft injection for internal nasal valve collapse. Ear Nose Throat J 86(5):272–273	Technique note/case report level; <10 patients; no validated outcome measure reported	Technique note/case report	PubMed
**Category E — Intervention relevance: nasal valve intervention not separable from concurrent nasal/sinonasal procedures, or NVD not a defined primary diagnosis (PRISMA exclusion criterion: Intervention relevance)**					
26	Stacey DH, Cook TA, Marcus BC (2009)	Correction of internal nasal valve stenosis: a single surgeon comparison of butterfly versus traditional spreader grafts. Ann Plast Surg 63(3):280–284	Comparator rhinoplasty study (n=82); NVD intervention not separable from concurrent rhinoplasty in all patients; no NOSE score; population mixes post-rhinoplasty and primary cases without isolable NVD subgroup	Retrospective comparative case series	PubMed
27	Agdogan Ö, Ersözlü T (2025)	Impact of scroll ligament preservation on nasal airway patency in rhinoplasty: rhinomanometry and acoustic rhinometry. Aesthetic Plast Surg 49(7):1857–1867	Rhinoplasty study focused on scroll ligament preservation; NVD not a defined primary diagnosis; valve dysfunction outcomes not isolable from the preservation technique	Retrospective cohort	PubMed
28	Palesy T et al. (2015)	Airflow and patient-perceived improvement following rhinoplastic correction of external nasal valve dysfunction. JAMA Facial Plast Surg 17(2):131–136	n=19; all patients received costal cartilage grafts with extensive concurrent sinonasal procedures at a tertiary centre; NVD-specific functional outcome not isolable. Borderline criterion D for NVD-pure subgroup.	Prospective case series (n=19)	PubMed
29	Bewick JC, Buchanan MA, Frosh AC (2013)	Internal nasal valve incompetence is effectively treated using batten graft functional rhinoplasty. Int J Otolaryngol 2013:734795	Alar batten graft series (n=107) for INVI; VAS outcomes only (no NOSE score); NVD defined by nasal strip test preoperatively only, not a validated diagnostic criterion	Retrospective case series (n=107)	PubMed
30	Millman B (2002)	Alar batten grafting for management of the collapsed nasal valve. Laryngoscope 112(3):574–579	Mixed case series of nasal valve surgery; NVD not separable from concurrent rhinoplasty procedures; non-validated subjective outcome scale	Retrospective case series	PubMed
31	O'Halloran LR (2003)	The lateral crural J-flap repair of nasal valve collapse. Otolaryngol Head Neck Surg 128(5):640–649	Technique description series; combined population undergoing concurrent procedures; NVD-specific functional outcomes not isolable	Retrospective case series	PubMed
32	Bloching MB (2007) — Breathe® implant series	Disorders of the nasal valve area: implant experience in 22 patients. GMS Curr Top Otorhinolaryngol Head Neck Surg 6:Doc07	Mixed aetiology (post-rhinoplasty, primary collapse, cleft nose); NVD-specific outcomes not separately reported; unvalidated satisfaction score only	Retrospective case series (n=22)	PubMed
33	Rujanavej V et al. (2012)	The validity of peak nasal inspiratory flow as a screening tool for nasal obstruction. J Med Assoc Thai 95(9):1205–1210	General nasal obstruction population including polyps, allergic rhinitis, turbinate hypertrophy, sinusitis; NVD not separately defined or analysable; reference standard is rhinomanometry, not NVD-specific diagnosis	Diagnostic accuracy cross-sectional	PubMed
**Category F — Wrong population or wrong outcomes: non-NVD population, no validated NVD-specific outcome data, duplicate/extension publications of already-included studies (PRISMA exclusion criterion: Population / Outcomes)**					
34	Wang Y, Bonaparte JP (2019)	Diagnosis and management of septal deviation and nasal valve collapse: a survey of Canadian otolaryngologists. J Otolaryngol Head Neck Surg 48(1):71	Survey of physician practice patterns; no clinical patient outcome data	Cross-sectional survey	PubMed
35	Clark DW et al. (2018)	Nasal airway obstruction: prevalence and anatomic contributors. Ear Nose Throat J 97(6):173–176	Epidemiological prevalence study; no NVD-specific diagnostic or surgical outcome data	Cross-sectional study	PubMed
36	Poirrier AL et al. (2014)	External nasal valve collapse: validation of novel outcome measurement tool. Rhinology 52(2):127–132	Tool validation study; primary outcome is inter-rater agreement of a scoring system, not diagnostic performance or treatment outcome for NVD	Validation study	PubMed
37	Sufyan AS et al. (2013)	Effects of alar batten grafts on nasal valve collapse and nasal steroid use. JAMA Facial Plast Surg 15(3):182–186	Concurrent allergic rhinitis population; primary outcome is steroid use reduction, not a validated functional NVD outcome; NVD and allergic contributions not separable	Prospective case series	PubMed
38	Wu J et al. (2024)	Radiographic study of the nasal valve in CT evaluation in Asian patients with unilateral cleft lip nose. Aesthetic Plast Surg 48(12):2412–2422	Cleft lip–nose deformity population; not NVD per inclusion criteria population definition	Retrospective CT study	PubMed
39	Ephrat M, Jacobowitz O, Driver M (2021)	Quality-of-life impact after in-office treatment of nasal valve obstruction with a radiofrequency device: 2-year results. Int Forum Allergy Rhinol 11(4):755–765	Follow-up extension of the Jacobowitz 2019 prospective cohort (already included as [60]); overlapping patient population; not an independent primary study	Prospective follow-up extension (n=39)	PubMed
40	Yao WC et al. (2023)	Two-year outcomes of temperature-controlled radiofrequency device treatment of the nasal valve. Laryngoscope Investig Otolaryngol 8(4):808–815	Follow-up extension of the Silvers 2021 RCT cohort (already included as [20]); overlapping patient population; not an independent primary study	Prospective follow-up (n=122, 2yr)	PubMed

	Author(s) & Year	Full Reference (abbreviated)	Reason for Exclusion	Study Design	DB
41	Stolovitzky P et al. (2018)	A prospective study for treatment of nasal valve collapse due to lateral wall insufficiency: outcomes using a bioabsorbable implant. Laryngoscope 128(11):2483–2489	6-month preliminary data from same multicenter LATERA cohort as Sidle 2020 (already included as [18]); overlapping patient population; duplicate publication of early subset data	Prospective multicenter (n=101, 6mo)	PubMed
42	Bikhazi N et al. (2021)	Bioabsorbable implant for treatment of nasal valve collapse with or without concomitant procedures. Facial Plast Surg 38(5):495–503	24-month analysis of overlapping LATERA multicenter cohorts (partially overlapping with Sidle 2020, included as [18]); mixed concurrent procedures; also duplicate population	Prospective multicenter post-market (n=277, 24mo)	PubMed
43	Adverse events associated with bioabsorbable nasal implants: MAUDE database analysis (2023)	Adverse events associated with bioabsorbable nasal implants: a MAUDE database analysis. Otolaryngol Head Neck Surg 169(1):191–194	Adverse event database analysis only; no functional NVD outcome data (NOSE/VAS); wrong outcome type per inclusion criteria	Regulatory database analysis	PubMed
44	Menger DJ et al. (2014)	Surgery of the external nasal valve: the correlation between subjective and objective measurements. Clin Otolaryngol 39(2):150–155	External valve surgical series; primary outcome is ROE score (rhinoplasty outcomes evaluation), not NOSE/VAS; ROE not a validated NVD-specific instrument per inclusion criteria	Retrospective case series	PubMed
45	Gillman GS et al. (2021)	Predictors of long-term NOSE score stability following septoplasty with inferior turbinate reduction. Laryngoscope 131(9):E2105–E2110	Septoplasty + inferior turbinate reduction cohort for septal deviation; NVD intervention explicitly excluded; cited for background NOSE score data only	Retrospective case series	PubMed
**Category G — General nasal obstruction population: diagnostic or surgical studies where NVD is not separately defined, diagnosed, or analysable as a subgroup (PRISMA exclusion criterion: Population)**					
46	Bermüller C et al. (2008)	Diagnostic accuracy of peak nasal inspiratory flow and rhinomanometry in functional rhinosurgery. Laryngoscope 118(4):605–610	General structural nasal stenosis population (septal deviation, turbinate hypertrophy, mixed); NVD not separately defined or analysable as a subgroup; reference standard is 'overall clinical judgment', not NVD-specific	Prospective diagnostic accuracy (n=93)	PubMed
47	Toyserkani NM, Frisch T, Von Buchwald C (2013)	Postoperative improvement in acoustic rhinometry after septoplasty correlates with long-term satisfaction. Rhinology 51(2):171–175	Septoplasty-only population; NVD not a defined or separately analysed diagnosis; acoustic rhinometry and NOSE correlation data used for background discussion only	Prospective cohort (septoplasty)	PubMed
48	Haavisto LE, Sipilä JI (2013)	Acoustic rhinometry, rhinomanometry and visual analogue scale before and after septal surgery: a prospective 10-year follow-up. Clin Otolaryngol 38(1):23–29	Septoplasty-only population; rhinometry used for general nasal obstruction; NVD not defined or separable as specific diagnosis; cited in Discussion for objective vs subjective mismatch only	Prospective 10-year follow-up (septoplasty)	PubMed
49	Edizer DT et al. (2013)	Nasal obstruction following septorhinoplasty: how well does acoustic rhinometry work? Eur Arch Otorhinolaryngol 270(2):609–613	Septorhinoplasty population for mixed cosmetic and functional indications; NVD not a defined primary diagnosis; acoustic rhinometry correlation studied in general post-rhinoplasty population	Prospective case series (septorhinoplasty)	PubMed
50	Vogt K et al. (2016)	Four-phase rhinomanometry: a multicentric retrospective analysis of 36,563 clinical measurements. Eur Arch Otorhinolaryngol 273(5):1185–1198	Normative/methodology data study across multiple indications; NVD-specific diagnostic accuracy not separately reported; used as background reference for rhinomanometry methodology	Retrospective multicentre methodology study	PubMed
51	Vogt K, Zhang L (2012)	Airway assessment by four-phase rhinomanometry in septal surgery. Curr Opin Otolaryngol Head Neck Surg 20(1):33–39	Narrative review of rhinomanometry in septal surgery; not an original clinical study; no NVD-specific primary data	Narrative review	PubMed
**Category H — Insufficient data: technique reports with < 10 NVD patients, non-validated outcome measures, or studies where functional NVD data are not extractable (PRISMA exclusion criterion: Small sample size / Wrong outcomes)**					
52	Maalouf R et al. (2016)	A functional tool to differentiate nasal valve collapse from other causes of nasal obstruction: the FRIED test. J Appl Physiol 121(3):343–347	Small diagnostic study; NVD-collapsed subgroup insufficient to reliably extract sensitivity/specificity per inclusion criteria; referenced for future research directions only	Diagnostic study (small NVD subgroup)	PubMed
53	Friedman M, Ibrahim H, Syed Z (2003)	Nasal valve suspension: an improved, simplified technique for nasal valve collapse. Laryngoscope 113(2):381–385	Technique modification report; n=7 patients with isolable suspension data; below ≥10 patient threshold	Retrospective case series (n<10)	PubMed
54	Reiffel AJ, Cross KJ, Spinelli HM (2010s)	Nasal spreader grafts: a comparison of Medpor to autologous tissue reconstruction	Alloplastic vs autologous spreader graft comparison in cosmetic rhinoplasty; NVD not a defined primary diagnosis; functional NVD outcomes not isolable from cosmetic rhinoplasty	Retrospective comparative	PubMed
55	Nasal patency after open rhinoplasty with spreader grafts (Pitanguy group, 2012)	Nasal patency after open rhinoplasty with spreader grafts. Braz J Otorhinolaryngol 78(2):126–131	Mixed cosmetic + functional rhinoplasty cohort (n=20); patients without functional complaint included; valve outcomes not separable from cosmetic rhinoplasty; non-NVD population per inclusion criteria	Prospective case series (n=20)	PubMed
56	André RF, Vuyk HD (2006)	Sub-alar batten grafts as treatment for nasal valve incompetence: description of technique and functional evaluation. Rhinology 44(2):118–122	Technique description with small patient series; primary outcome is technical description; NVD functional outcomes reported in <10 patients with isolable sub-alar batten graft alone	Technique description/case series (<10 NVD-pure)	PubMed
57	Ballert JA, Park SS (2006)	Functional rhinoplasty: treatment of the dysfunctional nasal sidewall. Facial Plast Surg 22(1):49–54	Technique review/small case series; no validated outcome measure (NOSE/VAS) reported; <10 patients with isolable NVD outcome data	Technique review/case series	PubMed
58	Bewick JC, Frosh AC (2006)	Functional rhinoplasty with batten and spreader grafts for correction of internal nasal valve incompetence. Rhinology 44(2):114–117	n=23 for INVI; VAS outcomes only (no NOSE score); NVD defined by clinical exam without validated tool; VAS for obstruction is a secondary outcome per inclusion criteria	Prospective series (n=23, VAS only)	PubMed
59	Mendelsohn M (2005)	Straightening the crooked middle third of the nose: using porous polyethylene extended spreader grafts. Arch Facial Plast Surg 7(2):74–80	Alloplastic (porous polyethylene) spreader graft technique in cosmetic rhinoplasty; NVD not a primary diagnosis; functional NVD outcomes not isolable from cosmetic procedure	Retrospective case series	PubMed

Exclusion criteria legend — A: Wrong publication type (narrative reviews, editorials, consensus statements, secondary systematic reviews/meta-analyses) | B: Non-clinical studies (cadaveric, anatomical, computational without clinical patient data) | C: Pediatric-only population (all patients < 18 yr) | D: Small sample size (< 10 patients) or no extractable NVD outcomes | E: NVD intervention not separable from concurrent nasal procedures | F: Wrong population or outcomes; duplicate or extension publications of already-included studies | G: General nasal obstruction population — NVD not separately defined or analysable | H: Technique reports with insufficient NVD patient data or non-validated outcomes

DB = primary database where study was identified (PubMed/MEDLINE, Embase, Cochrane Library, or Ref = reference list screening).
