# Supplementary material for: Diagnostic Approaches and Surgical Outcomes in Nasal Valve Dysfunction: A Systematic Review
Source: Diagnostics (Basel). 2026 Apr 28;16(9):1324. doi: 10.3390/diagnostics16091324 (PMC13164084; doi:10.3390/diagnostics16091324)
Supplement: Supplementary file 1 [file diagnostics-16-01324-s001.zip › Table S1 PRISMA 2020 Checklist.pdf]

PRISMA 2020 Checklist

"Diagnostic Approaches and Surgical Outcomes in Nasal Valve Dysfunction: A Systematic Review"

Diagnostics (MDPI) | Manuscript ID: diagnostics-4191253 | Revision 1

Corresponding author: Luana-Maria Gherasie | PROSPERO: CRD420261351284

Supplementary Materials attached: PRISMA 2020 for Abstracts Checklist (File 2) | Excluded Studies Table S1 | Risk of Bias Figure S1

Section and Topic	Item #	Checklist Item	Location in Manuscript
**TITLE**			
**Title**	1	Identify the report as a systematic review.	Title page, line 10: 'Diagnostic Approaches and Surgical Outcomes in Nasal Valve Dysfunction: A Systematic Review'
**ABSTRACT**			
**Abstract**	2	See the PRISMA 2020 for Abstracts checklist.	Lines 27–34 (Background, Methods, Results, Conclusions). See Supplementary File 2 — PRISMA 2020 for Abstracts Checklist. Funding line added at line 31.
**INTRODUCTION**			
**Rationale**	3	Describe the rationale for the review in the context of existing knowledge.	Section 1, lines 43–82: epidemiology of NVD, absence of gold-standard diagnostic, heterogeneity of treatment evidence. Sections 1.1–1.2 detail diagnostic and surgical context.
**Objectives**	4	Provide an explicit statement of the objective(s) or question(s) the review addresses.	Section 1.3, line 84: primary research question on diagnostic methods; secondary question on surgical outcomes and supporting evidence.
**METHODS**			
**Eligibility criteria**	5	Specify the inclusion and exclusion criteria for the review and how studies were grouped for the syntheses.	Section 2.3 and Table 2, lines 98–118: PICOS framework (population, study design, intervention, outcomes, publication characteristics); grouping into diagnostic vs. treatment synthesis stated in Section 2.9.
**Information sources**	6	Specify all databases, registers, websites, organisations, reference lists and other sources searched or consulted. Specify the date when each was last searched.	Section 2.4, lines 120–142: PubMed/MEDLINE, Embase, Cochrane Library; last search date January 2026; reference lists of included studies manually screened.
**Search strategy**	7	Present the full search strategies for all databases, registers and websites, including any filters and limits used.	Section 2.4, lines 130–142: full Boolean search strings for diagnostic and surgical outcome queries provided verbatim; language (English) and date (January 1990–January 2026) limits stated.
**Selection process**	8	Specify the methods used to decide whether a study met the inclusion criteria, including how many reviewers screened each record and each report retrieved, whether they worked independently, and details of automation tools used.	Section 2.5, lines 144–152: two independent reviewers; title/abstract screening followed by full-text eligibility assessment; disagreements resolved by consensus; PRISMA 2020 flow diagram (Figure 1).

Section and Topic	Item #	Checklist Item	Location in Manuscript
**Data collection process**	9	Specify the methods used to collect data from reports, including how many reviewers collected data from each report, whether they worked independently, any processes for obtaining data from investigators, and details of automation tools.	Section 2.6, lines 152–156: standardised data collection form; two independent reviewers; change scores calculated when not directly reported.
**Data items**	10a	List and define all outcomes for which data were sought. Specify whether all results compatible with each outcome domain were sought and if not, the methods used to decide which results to collect.	Section 2.7, lines 156–162: primary outcome ΔNOSE defined with MCID threshold; secondary outcomes listed (VAS, PNIF, rhinomanometry, acoustic rhinometry, complications, revision rates).
	10b	List and define all other variables for which data were sought (e.g. participant and intervention characteristics, funding sources). Describe assumptions about missing or unclear information.	Section 2.6, lines 152–156: study design, sample size, demographics, type of NVD, diagnostic modality or surgical technique, concomitant procedures, follow-up duration.
**Risk of bias assessment**	11	Specify the methods used to assess risk of bias in included studies, including details of tool(s) used, how many reviewers assessed each study and whether they worked independently.	Section 2.8, lines 166–168: RoB 2 for RCTs, ROBINS-I for observational/non-randomised studies, QUADAS-2 for diagnostic accuracy studies; two independent reviewers; discrepancies resolved through discussion.
**Effect measures**	12	Specify for each outcome the effect measure(s) used in the synthesis or presentation of results.	Section 2.9, lines 170–182: mean change in NOSE score (ΔNOSE) as primary effect measure; mean change in secondary outcome scores (VAS, PNIF). Random-effects DerSimonian–Laird model.
**Synthesis methods**	13a	Describe the processes used to decide which studies were eligible for each synthesis.	Section 2.9, lines 170–174: narrative synthesis for diagnostic modalities (heterogeneous reference standards); quantitative synthesis for surgical outcomes when ≥3 studies reported comparable NOSE data.
	13b	Describe any methods required to prepare the data for presentation or synthesis, such as handling of missing summary statistics or data conversions.	Section 2.9, lines 174–180: ΔNOSE calculated when not directly reported; SD of change estimated assuming r=0.5; variance derived from CI/p-values/SE when available; studies excluded from synthesis if variance unavailable.
	13c	Describe any methods used to tabulate or visually display results of individual studies and syntheses.	Section 2.9, line 180: 'Results of individual diagnostic studies were summarised in a structured table (Table 3). Results of individual surgical studies were visually represented in a forest plot (Figure 2). Narrative descriptions of individual study findings are provided in Section 3.5.'
	13d	Describe any methods used to synthesize results and provide a rationale for the choice(s). If meta-analysis was performed, describe the model(s), method(s) to identify heterogeneity, and software used.	Section 2.9, lines 172–182: random-effects DerSimonian–Laird model; I^2 statistic for heterogeneity; inverse variance weighting; RevMan and Stata software.

Section and Topic	Item #	Checklist Item	Location in Manuscript
	13e	Describe any methods used to explore possible causes of heterogeneity among study results.	Section 2.9, line 178 and Discussion lines 303–307: heterogeneity assessed via I^2; subgroup/meta-regression not feasible due to limited studies with homogeneous reporting; acknowledged as limitation.
	13f	Describe any sensitivity analyses conducted to assess robustness of the synthesized results.	Section 2.9, line 178: sensitivity analyses conducted by excluding studies at high risk of bias; results reported in Section 3.3, line 259.
**Reporting bias assessment**	14	Describe any methods used to assess risk of bias due to missing results in a synthesis (arising from reporting biases).	Section 2.9, lines 178–180: publication bias assessed qualitatively rather than by funnel plot (insufficient RCTs); acknowledged in Discussion line 303.
**Certainty assessment**	15	Describe any methods used to assess certainty (or confidence) in the body of evidence for an outcome.	Section 3.4, line 265: GRADE criteria applied; certainty rated low for diagnostic accuracy and low-to-moderate for surgical outcomes.
**RESULTS**			
**Study selection**	16a	Describe the results of the search and selection process, ideally using a flow diagram.	Section 3.1, lines 184–188 and Figure 1 (PRISMA 2020 flow diagram): 1,245 records identified → 163 full-text assessed → 72 studies included.
	16b	Cite studies that might appear to meet the inclusion criteria, but which were excluded, and explain why they were excluded.	Section 2.5, line 148: reasons for full-text exclusions stated (wrong population, non-clinical design, insufficient sample size, lack of relevant outcomes, inability to isolate NVD). Full list in Supplementary Table S1 (attached separately).
**Study characteristics**	17	Cite each included study and present its characteristics.	Table 1 (study characteristics overview); Sections 3.1.1–3.1.2, lines 190–218: study design, countries, sample sizes, follow-up, outcomes, intervention types.
**Risk of bias in studies**	18	Present assessments of risk of bias for each included study.	Section 3.4, lines 261–265: overall RoB described. Individual study-level assessments in Supplementary Figure S1 (attached separately). Critical appraisal per technique in Section 3.5.
**Results of individual studies**	19	For all outcomes, present for each study summary statistics for each group and an effect estimate and its precision, ideally using structured tables or plots.	Table 3 (diagnostic modality performance data); Figure 2 (forest plot with 95% CI for individual surgical studies); Section 3.5, lines 269–280: narrative critical appraisal per study/technique.
**Results of syntheses**	20a	For each synthesis, briefly summarise the characteristics and risk of bias among contributing studies.	Section 3.3, lines 255–260: 12 studies, ~1,210 patients; predominantly observational (Level IV); moderate-to-high risk of bias noted.
	20b	Present results of all statistical syntheses. If meta-analysis was done, present the summary estimate and its precision and measures of heterogeneity.	Section 3.3, lines 257–260: mean ΔNOSE 40–55 points; random-effects pooled estimate with 95% CI in Figure 2; I^2 reported (moderate-to-high).

Section and Topic	Item #	Checklist Item	Location in Manuscript
	20c	Present results of all investigations of possible causes of heterogeneity among study results.	Section 3.3, line 259: heterogeneity attributed to variability in patient selection, surgical technique, and follow-up duration; formal subgroup/meta-regression not feasible (acknowledged as limitation, Discussion line 303).
	20d	Present results of all sensitivity analyses conducted to assess robustness of the synthesized results.	Section 3.3, line 259: sensitivity analysis excluding high-risk-of-bias studies did not materially alter direction or magnitude of effect.
**Reporting biases**	21	Present assessments of risk of bias due to missing results for each synthesis assessed.	Section 3.4, line 263 and Discussion line 303: publication bias assessed qualitatively; positive-result bias acknowledged given predominance of uncontrolled case series.
**Certainty of evidence**	22	Present assessments of certainty (or confidence) in the body of evidence for each outcome assessed.	Section 3.4, line 265: GRADE applied; low certainty for diagnostic accuracy; low-to-moderate certainty for surgical outcomes.
**DISCUSSION**			
**Discussion**	23a	Provide a general interpretation of the results in the context of other evidence.	Section 4 (Discussion), lines 281–308: synthesis of diagnostic and surgical findings; comparison with existing literature and clinical consensus statements; proposed diagnostic algorithm (Figure 3).
	23b	Discuss any limitations of the evidence included in the review.	Section 4, lines 303–308: predominance of Level IV observational studies; publication bias; inability to isolate valve-specific intervention effects; heterogeneous outcome reporting.
	23c	Discuss any limitations of the review processes used.	Section 4, lines 303–308: subgroup/meta-regression not feasible; English-language restriction; grey literature not searched; some included studies did not specify NVD definition.
	23d	Discuss implications of the results for practice, policy, and future research.	Section 4, lines 295–308: five clinical recommendations; multimodal diagnostic algorithm (Figure 3); call for RCTs comparing techniques; 5–10-year follow-up studies needed.
**OTHER INFORMATION**			
**Registration and protocol**	24a	Provide registration information for the review, including register name and registration number, or state that the review was not registered.	Section 2.2, line 96: registered in PROSPERO, registration number CRD420261351284.
	24b	Indicate where the review protocol can be accessed, or state that a protocol was not prepared.	Section 2.2, line 96: predefined protocol registered in PROSPERO prior to study selection; accessible at www.crd.york.ac.uk/prospero.
	24c	Describe and explain any amendments to information provided at registration or in the protocol.	Section 2.2, line 96: 'No amendments were made to the registered protocol subsequent to study commencement.'

Section and Topic	Item #	Checklist Item	Location in Manuscript
**Support**	25	Describe sources of financial or non-financial support for the review, and the role of the funders or sponsors in the review.	Funding section, line 323: 'This research received no external funding.' Also stated in Abstract, line 31.
**Competing interests**	26	Declare any competing interests of review authors.	Conflicts of Interest section, line 331: 'The authors declare no conflicts of interest.'
**Availability of data, code and other materials**	27	Report which of the following are publicly available and where they can be found: template data collection forms; data extracted from included studies; data used for all analyses; analytic code; any other materials used in the review.	Data Availability Statement, line 321: standardised data extraction template, extracted datasets, and statistical analysis code (RevMan/Stata) available from corresponding author on request. PRISMA flow diagram and risk-of-bias summaries provided as Supplementary Materials.

From: *Page MJ, McKenzie JE, Bossuyt PM, Boutron I, Hoffmann TC, Mulrow CD, et al. The PRISMA 2020 statement: an updated guideline for reporting systematic reviews. BMJ 2021;372:n71. doi: 10.1136/bmj.n71.* *Licensed under CC BY 4.0.*
