# Supplementary figures and images for: Diagnostic Approaches and Surgical Outcomes in Nasal Valve Dysfunction: A Systematic Review
Source: Diagnostics (Basel). 2026 Apr 28;16(9):1324. doi: 10.3390/diagnostics16091324 (PMC13164084; doi:10.3390/diagnostics16091324)

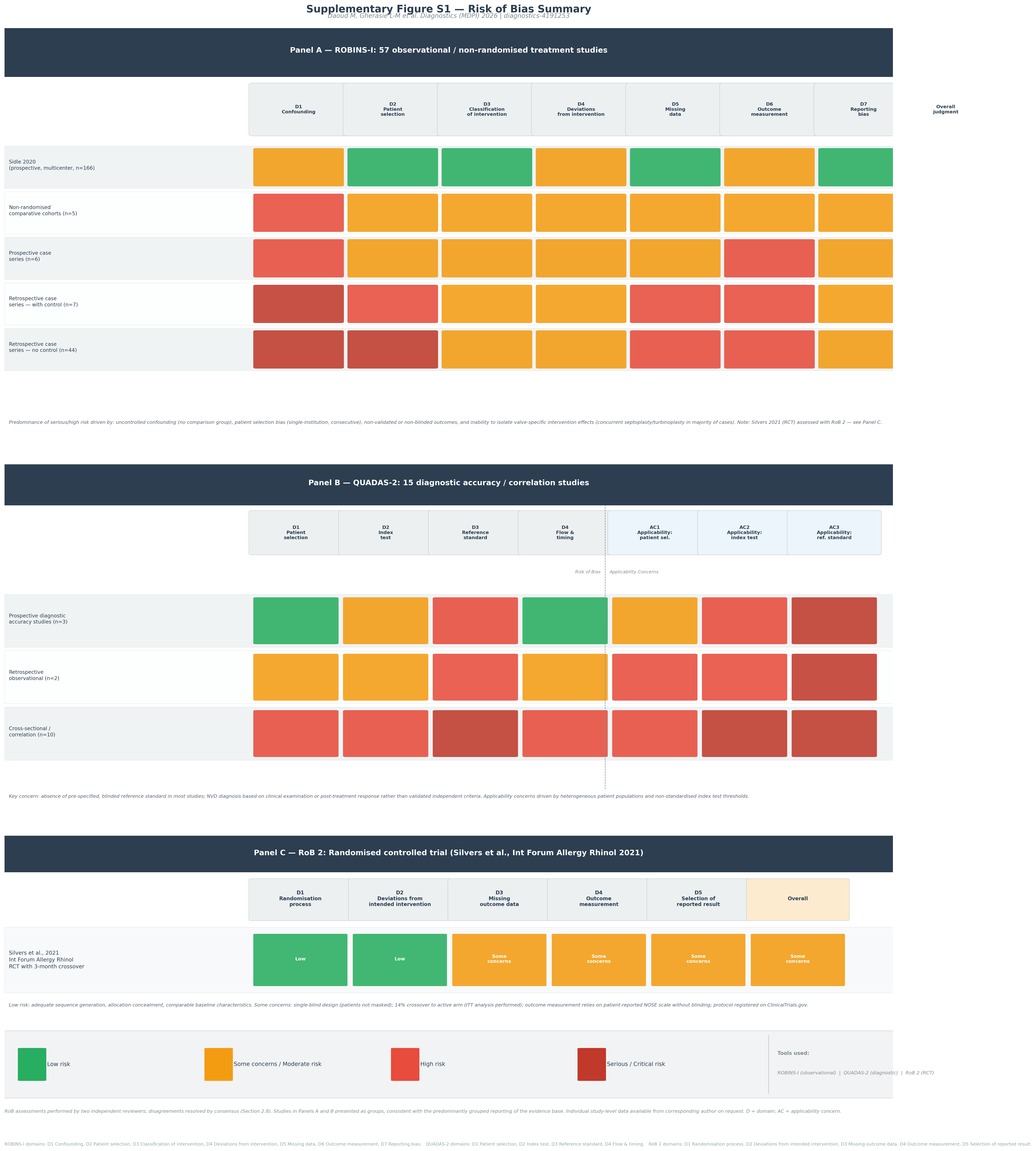

Supplement: Supplementary file 1 [file diagnostics-16-01324-s001.zip › Figure S1 Risk of Bias Summary.png]
